# Proteomic analysis of endothelial cold-adaptation

**DOI:** 10.1186/1471-2164-12-630

**Published:** 2011-12-22

**Authors:** Michael AJ Zieger, Mahesh P Gupta, Mu Wang

**Affiliations:** 1 Methodist Research Institute, Indiana University Health, Indianapolis, IN 46202, USA; 2 Department of Medicine, Indiana University School of Medicine, Indianapolis, IN 46202, USA; 3 Department of Biochemistry and Molecular Biology, Indiana University School of Medicine, Indianapolis, IN 46202, USA

## Abstract

**Background:**

Understanding how human cells in tissue culture adapt to hypothermia may aid in developing new clinical procedures for improved ischemic and hypothermic protection. Human coronary artery endothelial cells grown to confluence at 37°C and then transferred to 25°C become resistant over time to oxidative stress and injury induced by 0°C storage and rewarming. This protection correlates with an increase in intracellular glutathione at 25°C. To help understand the molecular basis of endothelial cold-adaptation, isolated proteins from cold-adapted (25°C/72 h) and pre-adapted cells were analyzed by quantitative proteomic methods and differentially expressed proteins were categorized using the DAVID Bioinformatics Resource.

**Results:**

Cells adapted to 25°C expressed changes in the abundance of 219 unique proteins representing a broad range of categories such as translation, glycolysis, biosynthetic (anabolic) processes, NAD, cytoskeletal organization, RNA processing, oxidoreductase activity, response-to-stress and cell redox homeostasis. The number of proteins that decreased significantly with cold-adaptation exceeded the number that increased by 2:1. Almost half of the decreases were associated with protein metabolic processes and a third were related to anabolic processes including protein, DNA and fatty acid synthesis. Changes consistent with the suppression of cytoskeletal dynamics provided further evidence that cold-adapted cells are in an energy conserving state. Among the specific changes were increases in the abundance and activity of redox proteins glutathione S-transferase, thioredoxin and thioredoxin reductase, which correlated with a decrease in oxidative stress, an increase in protein glutathionylation, and a recovery of reduced protein thiols during rewarming from 0°C. Increases in S-adenosylhomocysteine hydrolase and nicotinamide phosphoribosyltransferase implicate a central role for the methionine-cysteine transulfuration pathway in increasing glutathione levels and the NAD salvage pathway in increasing the reducing capacity of cold-adapted cells.

**Conclusions:**

Endothelial adaptation to mild-moderate hypothermia down-regulates anabolic processes and increases the reducing capacity of cells to enhance their resistance to oxidation and injury associated with 0°C storage and rewarming. Inducing these characteristics in a clinical setting could potentially limit the damaging effects of energy insufficiency due to ischemia and prevent the disruption of integrated metabolism at low temperatures.

## Background

Hypothermia is utilized in several clinical situations. Moderate-to-deep hypothermia (32°-15°C) is the primary method for delaying ischemic injury in patients during cardiovascular [1,2], neurovascular [3] or trauma surgery [4]. More severe hypothermia (< 10°C) is used to protect isolated tissues and organs for transplantation. Therapeutic mild hypothermia (34°-32°C) improves the neurological recovery of patients following a cardiac arrest [5,6], stroke [7], or traumatic brain injury [8]. In each situation, the reduction in temperature decreases the metabolic demand for energy and slows the progression of injury. However, hypothermia also disrupts metabolic integration and impairs important functional processes, particularly when it is severe. For example, cold-induced vascular injury is characterized by a loss of endothelial cell-cell contact [9,10], a release of inflammatory cytokines [11,12], an increase in the expression of adhesion molecules [11,12], impaired vasoactivity [13,14] and cell death [15]. Unfortunately, both the protective and harmful mechanisms induced by hypothermia vary with temperature and are poorly understood and therefore its clinical utility remains limited.

Many organisms, from prokaryotes to plants and animals adapt to a range of temperatures and remain viable. Poikilotherms and mammalian hibernators adapt to cold and become resistant to injury from more prolonged and severe hypothermia and ischemia. Interestingly, the cells of non-hibernating mammals adapt to mild-moderate hypothermia in tissue culture [16-19] in what is likely a conserved response to cold and several mechanisms of cold-induced changes in gene expression have been identified. These include a generalized inhibition of transcription and translation, an increase in transcription of some RNA-binding chaperones, alternative splicing of pre-mRNAs and preferential translation of mRNAs that have cold-inducible internal ribosome entry sites (IRESs) [19]. In our studies of cold-adaptation, human coronary artery endothelial cells (HCAECs) cultured at 25°C become progressively more resistant over time to 0°C-injury and in particular to the oxidative stress induced by exposure to 0°C and rewarming [16]. The molecular basis of the adaptation remains largely unknown but the resulting protection at 0°C is due, in part, to the sequestration of catalytically active iron [16]. The protection may also be associated with an increase in intracellular glutathione, an important antioxidant and signaling molecule of the cell, at 25°C. Glutathione (GSH) reacts directly with free radicals, participates in the reductive detoxification of hydrogen peroxide and organic peroxides, serves as a co-factor in the enzymatic breakdown of xenobiotics and reacts with protein thiols to form mixed disulfides (P-SSG) under conditions of mild oxidative stress [20]. Protein glutathionylation is a reversible modification that provides a mechanism for protecting proteins from irreversible oxidative damage and for regulating protein function and thereby many diverse cellular processes such as cytoskeletal organization, ionic homeostasis and the expression of genes involved in antioxidant defenses [21]. Understanding the process of cold-adaptation and the characteristics of the protected state in human cells may be beneficial to the development of new clinical strategies for improved ischemic and hypothermic protection.

The aim of the present study was to characterize the cold-adapted state and the potential role of GSH in endothelial protection at 0°C. We performed quantitative proteomic analyses of human coronary artery endothelial cells collected before and after 72 h of culture at 25°C and classified the changes in protein abundance using the DAVID Bioinformatics Resource [22,23]. The most significant biological processes associated with cold-adaptation were translation, glycolysis, RNA processing, actin filament-based processes, and mRNA metabolic processes. Evidence of a regulated decrease in energy-consuming processes such as protein, DNA and fatty acid synthesis and suppression of cytoskeletal dynamics suggest that the cold-adapted state is hypometabolic. There was also evidence of a complementary increase in antioxidant protection as there were significant changes in categories relevant to GSH such as oxidoreductase activity and cell redox homeostasis. Among the many changes in cold-adapted endothelial cells were increases in the abundance of thioredoxin, thioredoxin reductase and glutathione S-transferase, which correlated with increases in their respective activities, increases in protein glutathionylation, recovery of reduced protein thiols and diminished oxidative stress following rewarming from 0°C.

## Results

### Analysis of changes in protein expression at 25°C

In order to characterize further the array of proteins altered during endothelial adaptation to cold, we used a label-free liquid chromatography/mass spectrometry (LC/MS)-based method [24] to study protein extracts from HCAECs cultured at 37°C or cells subsequently cold-adapted to 25°C for 72 h as this length of exposure to 25°C markedly increases GSH levels in cells and protects from 0°C cold exposure [16]. A total of 1089 proteins were identified (summary: Table 1; complete list of proteins: Additional file 1) and the number of proteins that decreased in abundance exceeded the number that increased by about 2:1 (202 decreased:102 increased). Priority 1 or 2 proteins were selected for further analysis and 181 of the 219 protein identifiers were successfully converted from the International Protein Index [25] to UniProt accession numbers. The assignment of protein priority is based on the peptide identification confidence from MS/MS data, which was described in detail previously [24,26].

**Table 1:** Changes in protein expression following cold-adaptation as determined by LC/MS-based label-free protein quantitative analysis.

Protein priority	Peptide ID confidence	Multiple sequences	Number of proteins	Number significant changes	Maximum absolute fold change	Number increasing at 25°C	Number decreasing at 25°C
1	High	Yes	440	169 (15.5%)	1.70	73	96
2	High	No	364	50 (4.6%)	1.92	12	38
3	Moderate	Yes	7	4 (0.04%)	1.23	0	4
4	Moderate	No	278	81 (7.4%)	2.37	17	64
Overall			1089	304 (27.9%)	2.37	102 (9.4%)	202 (18.5%)

A total of 1089 proteins were identified by LC/MS and 27.9% of these had significantly increased or decreased in abundance following 25°C/72 h. Priorities 1-4 were assigned based on confidence in peptide identification (High and Moderate) and whether or not there are multiple amino acid sequences (Yes or No) identified. Please see references [24] and [26] for more details about how the protein priority is determined.

**Figure 1:**
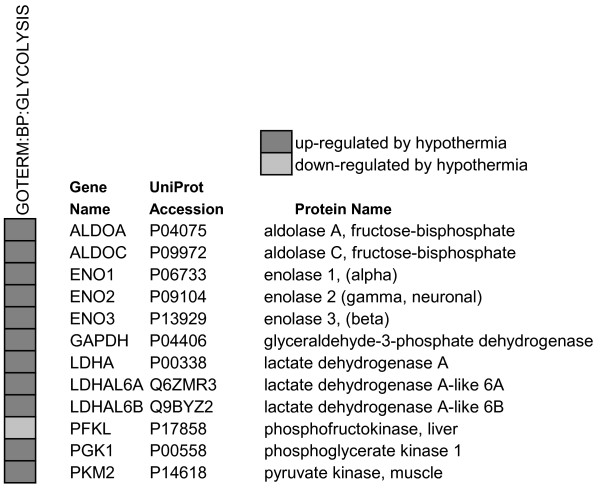
**Glycolytic proteins that were significantly up- or down-regulated at 25°C**.

To aid in the interpretation of the experimental findings, the accession numbers were uploaded to the DAVID Bioinformatics Resources website [22,23] and proteins were categorized by Gene Ontology (GO) biological process, molecular function or cellular component terms [27]. Twenty-four representative categories of interest are shown in Table 2 (see Additional file 2 for the complete set of categories). A large number of proteins that were up- or down-regulated by hypothermia are involved in primary metabolism (anabolic and catabolic processes), macromolecular metabolism, gene expression, biosynthesis (anabolic processes), protein metabolic processes and translation (56.7, 48.9, 34.8, 33.7, 31.5 and 22.5% of the total number of proteins that were analyzed, respectively). For these processes, the number of proteins that decreased exceeded the number that increased by ratios of 19-to-1 (translation), 8.4-to-1 (protein metabolism), 4.2-to-1 (gene expression), 3.6-to-1 (biosynthetic processes), 3-to-1 (macromolecular metabolism), and 2-to-1 (primary metabolic process). In addition, the categories of macromolecular complex assembly, ribonucleoprotein complex biogenesis, RNA processing, messenger RNA metabolic process, intracellular transport, protein folding, unfolded protein binding, microtubule cytoskeleton and response to stress also have more proteins that decreased in abundance than increased. Some processes show a net increase in proteins at 25°C. For example, for the catabolic process, the number of proteins that increased exceed the number that decreased by a ratio of 2.5-to-1. Twelve of the 21 catabolic proteins are also classified as glycolytic proteins, of which 11 *increased *and 1 decreased significantly at 25°C (Figure 1). GO categories such as cell proliferation, anti-apoptosis, cytoskeletal organization, actin filament-based processes, oxidoreductase activity and cell redox homeostasis also contain a greater number of protein increases than decreases.

**Table 2:** Selected GO^a ^biological process classifications of proteins differentially expressed in HCAECs following cold-adaptation.

# of Proteins						
UP	DN	Ratio^b^	%^c^	Gene ontology categories	P-Value^d^	Fold^e^
34	67	-2.0	56.7	Primary metabolic process	1.4 × 10^-3^	1.1
22	65	-3.0	48.9	Macromolecule metabolic process	1.9 × 10^-3^	1.3
12	50	-4.2	34.8	Gene expression	3.0 × 10^-6^	1.7
13	47	-3.6	33.7	Biosynthetic process	1.7 × 10^-3^	1.4
15	6	2.5	11.8	Catabolic process	9.9 × 10^-2^	1.4
11	1	11.0	6.7	Glycolysis	5.1 × 10^-12^	21.6
						
7	59	-8.4	31.5	Protein metabolic process	3.9 × 10^-5^	1.7
2	16	-8.0	10.1	Macromolecular complex assembly	2.2 × 10^-3^	2.3
0	11	- ∞	6.2	Ribonucleoprotein complex assembly	5.3 × 10^-5^	5.2
9	18	-2.0	15.2	RNA processing	1.1 × 10^-9^	4.2
8	11	-1.4	10.7	mRNA metabolic process	3.6 × 10^-7^	4.3
2	38	-19.0	22.5	Translation	2.8 × 10^-28^	10.2
5	11	-2.2	9.0	Intracellular transport	1.1 × 10^-2^	2.1
0	7	- ∞	3.9	Protein folding	1.8 × 10^-2^	3.3
0	9	- ∞	5.1	Unfolded protein binding^f^	5.0 × 10^-5^	6.9
						
6	5	1.2	6.2	Cell proliferation	3.3 × 10^-2^	2.1
6	1	6.0	3.9	Anti-apoptosis	3.5 × 10^-2^	2.9
3	3	1.0	3.4	Structure-specific DNA binding	2.5 × 10^-2^	3.6
						
11	7	1.6	10.1	Cytoskeletal organization	1.5 × 10^-5^	3.5
13	4	3.2	9.6	Actin filament-based process	2.5 × 10^-8^	6.0
1	11	-11.0	6.7	Microtubule cytoskeleton^g^	3.3 × 10^-2^	3.0
						
9	5	1.8	7.9	Oxidoreductase activity^f^	5.1 × 10^-2^	1.8
3	2	1.5	2.8	Cell redox homeostasis	6.4 × 10^-3^	6.7
11	16	-1.5	15.2	Response-to-stress	8.9 × 10^-2^	1.4

Proteins were classified using the Gene Functional Annotation Tool of DAVID bioinformatics resources. a. Gene Ontology; b. Ratios are negative where the number of proteins down-regulated at 25°C (# DN) > the number of proteins up-regulated at 25°C (# UP) and positive where # UP > # DN; c. % = the number of proteins in this category out of 178 proteins; d. Modified Fisher Exact P-value (probability of obtaining this many differentially expressed proteins in the category by chance); e. Fold-enrichment; f. GO *Molecular Function *term. g. GO *Cellular Component *term.

Two parameters used in DAVID, *P-value *and *fold-enrichment*, quantify the extent to which a given category is overrepresented within the dataset when compared to the precentage of similarly categorized genes from the entire genome. Categories with smaller P-values (< 0.05) and larger fold-enrichments (≥ 1.5) are considered to be of greater interest [23]. The most enriched groups from Table 2 are translation, glycolysis, RNA processing, actin filament-based processes and mRNA metabolic processes (P-values of 2.8 × 10^-28^, 5.1 × 10^-12^, 1.1 × 10^-9^, 2.5 × 10^-8 ^and 3.6 × 10^-7^, and fold-enrichment of 10.2, 21.6, 4.2, 6.0, and 4.3, respectively; see Table 2). Categories that may be relevant to GSH activity, such as oxidoreductase activity, response to stress and cell redox homeostasis appeared to be less enriched and of lower significance (fold-enrichment of 1.8, 1.4, and 6.7 and P-values of 5.1 × 10^-2^, 8.9 × 10^-2 ^and 6.4 × 10^-3^, respectively) and therefore potentially of reduced importance. These categories show mixed changes in protein abundance, with increase-to-decrease ratios of 1.8-to-1, 1-to-1.5, and 1.5-to-1.

### Oxidative stress and protein oxidation/reduction

**Figure 2:**
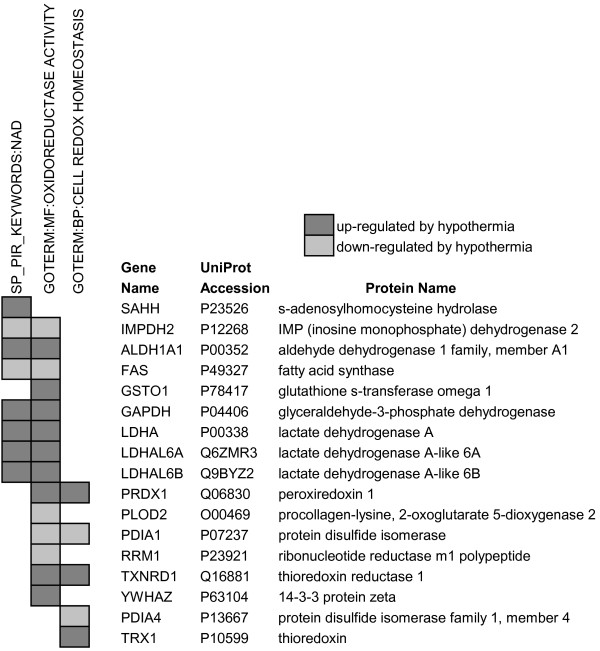
**Proteins that participate in redox reactions and were significantly up- or down- regulated at 25°C**.

Figure 2 lists proteins that have oxidoreductase activity or regulate cell redox homeostasis and were significantly up- or down-regulated at 25°C. Thioredoxin (TRX1), thioredoxin reductase 1 (TXNRD1), peroxiredoxin-1 (PRDX1) and glutathione S-transferase (GST, omega 1) increased in abundance and protein disulfide isomerases 1 and 4 (PDIA1, PDIA4) decreased significantly. TRX1 is an important cytoplasmic protein that reduces oxidized proteins such as peroxiredoxins and ribonucleotide reductase [28]. Thioredoxins (Trx) are reduced by thioredoxin reductases (TrxR) with reducing equivalents from NADPH in a manner analogous to the reduction of oxidized to reduced glutathione by GSSG reductase and NADPH. Peroxiredoxins play a protective role in cells by eliminating peroxides [28] and GSTs are important for detoxifying the products of oxidative stress, such as oxidized proteins, DNA or lipids, through their conjugation with GSH [20]. PDIA's 1 and 4 are found in the endoplasmic reticulum (ER) and are involved in the oxidative folding of nascent proteins by inducing the formation of intramolecular disulfide bonds [29]. The proteomic changes indicate that hypothermic adaptation potentially increases the cell's capacity to reduce oxidized proteins.

**Figure 3:**
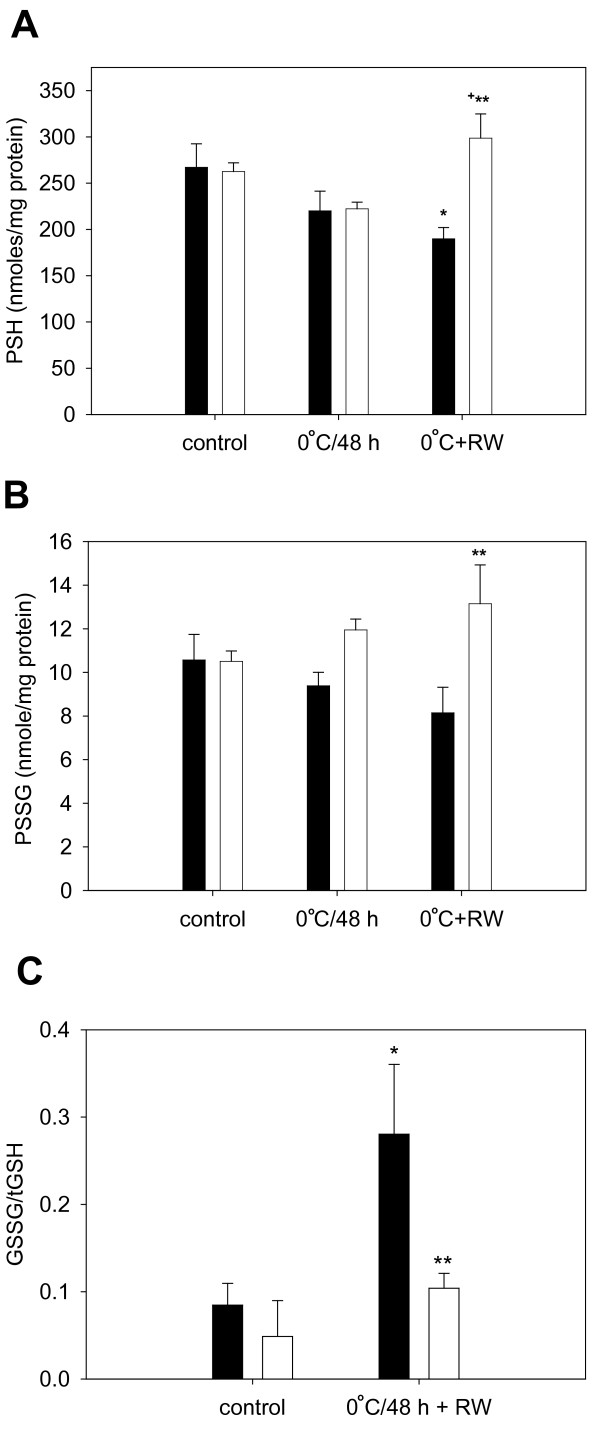
**Effect of cold-adaptation (25°C/72 h) and cold storage (0°C/48 h) on protein and non-protein thiols**. (A) Cold-adaptation prevents the significant loss of protein thiols (PSH) that occurs during cold storage/rewarming (*P < 0.01 vs. 37°C control; ** *P *< 0.001 vs. 37°C+0°C+RW; n = 5 experiments), possibly by the reduction of oxidized proteins during the rewarming phase (+*P *< 0.05 vs. 25°C+0°C; n = 5 experiments). (B) Cold-adapted cells have a significantly higher level of glutathiolated protein (PSSG) than non-adapted cells (37°C) following cold storage/rewarming (**P < 0.05 vs. 37°C+0°C+RW; n = 5 experiments). (C). Cold-adaptation attenuates the increase in GSSG/tGSH, indicative of oxidative stress, that occurs during cold storage/rewarming (*P < 0.001 vs. 37°C control; **P < 0.001 vs. 37°C+0°C+RW; n = 3 experiments). Open bars represent cell cold-adapted at 25°C for 72 h; closed bars represent cells maintained at 37°C for 72 h.

To determine if cold-adapted cells have an increased capacity to reduce oxidized proteins, confluent endothelial cells were cultured at 25°C for 72 h or maintained at 37°C (controls) and protein thiols (PSH), protein glutathionylation (PSSG) and free glutathione (oxidized, GSSG and total, tGSH) was measured immediately before or after exposure of cells to 0°C and subsequent rewarming. HCAECs are strongly susceptible to oxidative injury induced by 0°C exposure and rewarming [16]. PSH and PSSG in cells was unchanged after 72 h at 25°C (Figures 3A, B) as the treatment did not induce changes in GSSG/tGSH (Figure 3C). Exposure of control cells to 0°C followed by rewarming to 37°C for 3 h, however, decreased PSH (*P < 0.01; Figure 3A) and increased GSSG/tGSH (*P < 0.001 Figure 3C), which is indicative of oxidative stress. Similarly, cells treated with the pro-oxidant diamide at 37°C (1 mM/30 min) showed a significant decrease in PSH and an increase in GSSG/tGSH (data not shown). However, PSH in cold-adapted cells was significantly greater than in non-adapted cells following rewarming from 0°C (**P < 0.001 vs. 37°C+0°C+RW; Figure 3A). This may be indicative of enzymatic reduction of oxidized protein thiols during rewarming as PSH increased significantly during this phase (+*P *< 0.05 vs. 25°C+0°C; Figure 3A). PSSG trended lower with 0°C storage and 0°C plus rewarming but not in cold-adapted cells, which trended oppositely (*P < 0.05 vs. 37°C+0°C+RW; Figure 3B). Thus, cold-adapted cells have an increased capacity to recover PSH following cold-storage and rewarming when compared to non-adapted cells as well as an increased ability to glutathionylate proteins, which correlates with the observed increase in GST.

**Figure 4:**
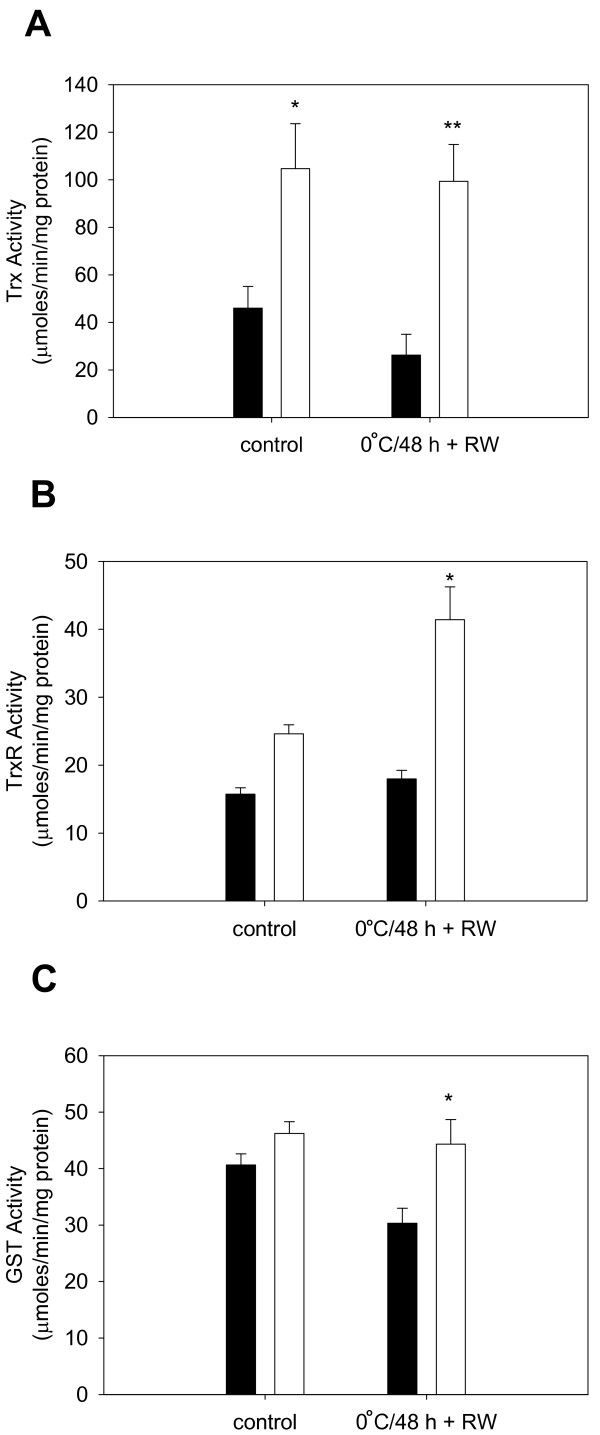
**Effect of cold-adaptation (25°C/72 h) and cold storage (0°C/48 h) on thioredoxin (Trx), thioredoxin reductase (TrxR) and GST (glutathione S transferase) activity**. (A) Cold-adaptation increases Trx activity before or after 0°C storage (*P < 0.05 vs. 37°C control; **P < 0.001 vs. 37°C + 0°C/48 h + RW; n = 5 experiments). (B) TrxR activity is greater in cold-adapted cells following 0°C storage and rewarming (*P < 0.05 vs. 37°C + 0°C/48 h + RW; n = 4 experiments). (C) GST activity in cold-adapted cells following 0°C storage and rewarming exceeds that of control cells (*P < 0.05 vs. 37°C + 0°C/48 h + RW; n = 4 experiments). Open bars represent cell cold-adapted at 25°C for 72 h; closed bars represent cells maintained at 37°C for 72 h.

To determine if the Trx-TrxR and GSH-GST systems are functionally more active in cold-adapted endothelial cells, Trx, TrxR and GST activities were measured in cells adapted to 25°C for 72 h and in 37°C control cells before and following 0°C storage plus rewarming. Figure 4A shows there is a significant increase in Trx activity with cold-adaptation (*P < 0.05 vs. 37°C control) and a significantly higher level of Trx and TrxR activity following rewarming from 0°C when compared to cold-stored control cells (**P < 0.01 vs. 37°C + 0°C/48 h + RW, Figure 4A and *P < 0.05 vs. 37°C + 0°C/48 h + RW, Figure 4B). This correlates with the increased recovery of PSH in cold-adapted cells following rewarming from 0°C (Figure 3A). GSH-dependent GST activity is also increased in cold-adapted cells following 0°C storage and rewarming when compared to cold-stored non-adapted cells (*P < 0.05 vs. 37°C + 0°C/48 h + RW; Figure 4C), which correlates with the increase in PSSG (Figure 3B) and tGSH observed following rewarming from 0°C [16]. In summary, the findings indicate that the increase in Trx-TrxR and GSH-GST activities may be an important component of the antioxidant protection of cold-adapted endothelial cells and their proteins during 0°C storage and rewarming.

### NAD metabolic proteins

The molecule nicotinamide adenine dinucleotide or NAD plays a central role in linking the energy producing catabolic reactions of the cell with the maintenance of redox homeostasis [30]. Significant changes in NAD metabolic proteins were identified in this study. Cells adapted to 25°C had an increase in the level of nicotinamide phosphoribosyltransferase (Nampt), the rate-limiting enzyme in NAD biosynthesis from nicotinamide [31] (see Additional file 1). There was also an increase in glycolytic enzymes that use NAD as an electron acceptor or cofactor. Glyceraldehyde-3-phosphate dehydrogenase (GAPDH), which reduces NAD^+ ^to NADH, increased at 25°C, as did lactate dehydrogenase (LDHA, LDHAL6A and LDHAL6B), which reduces pyruvate to lactate and regenerates NAD^+^(Figure 2). Interestingly, cold-adaptation increased the abundance of S-adenosylhomocysteine hydrolase (SAHH), an NAD^+ ^dependent enzyme and component of the methionine-cysteine transulfuration pathway that generates cysteine used to synthesize GSH [32]. Inosine monophosphate dehydrogenase (IMPDH2), the NAD^+^-dependent and rate-limiting enzyme of *de novo *guanine nucleotide synthesis [33], decreased at 25°C.

The phosphorylated and reduced form of NAD, NADPH, provides the reducing equivalents for GSH and thioredoxin reduction that bolsters the cell's resistance to oxidative stress. Several NADP(H)-dependent enzymes changed significantly in abundance during cold-adaptation. Aldehyde dehydrogenase (ALDH1A1), an NADP^+^-dependent enzyme, increased at 25°C (Figure 2). Its function in the cell is to oxidize toxic aldehydes produced during lipid peroxidation and thereby prevent their adduct formation with proteins or DNA [34]. Conversely, several proteins decreased in abundance, including ribonucleotide reductase (RRM1), a thioredoxin-dependent enzyme [35] that catalyzes the formation of deoxyribonucleotide from ribonucleotide [36]; and fatty acid synthase (FAS), which catalyzes the formation of long-chain fatty acids from acetyl CoA, malonyl CoA and NADPH.

### Proteins that regulate gene expression

**Figure 5:**
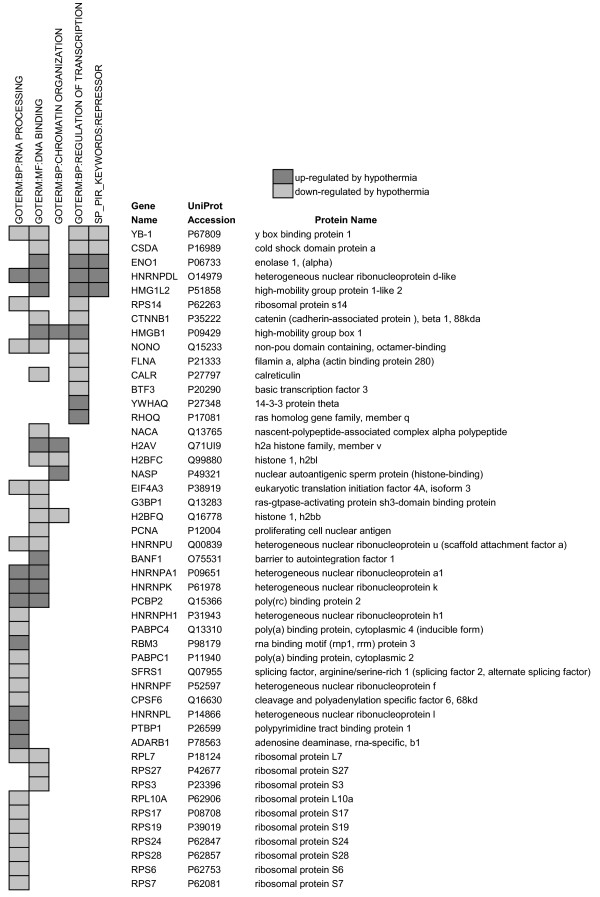
**Proteins that may regulate gene expression and were significantly up- or down-regulated at 25°C**.

Cold-adaptation induced significant changes in the abundance of proteins that organize chromatin, bind to RNA and DNA, and regulate transcription and translation (Figure 5). There were changes in the abundance of histone proteins (H2AV, H2BFC, H2BFQ), non-histone chromatin-binding proteins (HMGB1, HMG1L2, BANF1) and the histone chaperone NASP at 25°C. Two proteins that increased in abundance at 25°C induce transcriptional repression (ENO1 and HNRNPDL) and 2 cold shock domain proteins (YB-1, CSDA), which repress growth factor and stress response genes [37,38], decreased at 25°C. Another transcription factor that decreased at 25°C, β-catenin, binds to and regulates ROS-sensitive transcription factors such as HIF-1 and FOXO [39]. There was also a decrease in 11 ribosomal proteins and proteins associated with transcription initiation (BTF3), DNA replication/repair (PCNA), pre-mRNA processing (NONO, SRFS1, HNRNPH1, HNRNPF, CPSF6), mRNA elongation (HNRNPU), nonsense-mediated mRNA decay (EIF4A3), stress granule assembly (G3BP1, G3BP2) [40], translation regulation (RPS14, PABPC4, PABPC1) and targeting of nascent proteins to the endoplasmic reticulum (NACA). Conversely, there was a significant increase in RNA-binding proteins (PTBP1, HNRNPL and ADARB1), which process pre-mRNA, and HNRNPK, HNRNPA1 and PCBP2, which are multi-functional proteins involved in such processes as chromatin remodeling, transcriptional repression/activation, mRNA export, stabilization and turnover, and translational silencing/activation [41]. RBM3, a cold-inducible RNA-binding protein that is believed to enhance global protein synthesis [42], increased significantly at 25°C.

### Response to stress proteins

**Figure 6:**
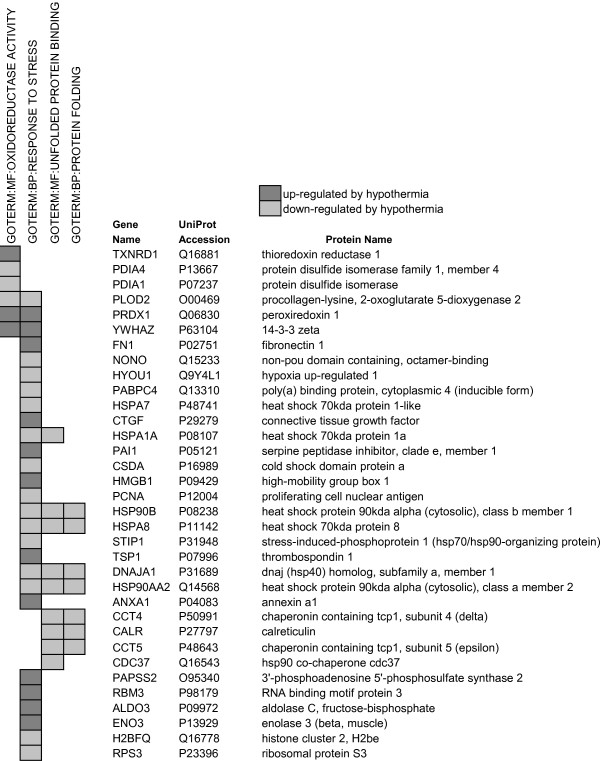
**Response-to-stress proteins that were significantly up- or down-regulated at 25°C**. (Note: the complete list for Oxidoreductase Activity is in Figure 2).

Figure 6 lists 27 *response-to-stress *proteins that were significantly up- or down-regulated with cold-adaptation. Eight are molecular chaperones or chaperone-associated proteins that assist with protein folding or macromolecular assembly and all were down-regulated (HYOU1, HSPA7, HSPA1A, HSP90B, HSPA8, STIP1, DNAJA1, and HSP90AA2). Other proteins that play a role in protein folding decreased in abundance, including PDIA1 and PDIA4, chaperonins CCT4 and CCT5, the co-chaperone CDC37 and the calcium-binding chaperone CALR. Five of the proteins listed in Figure 6 are ER-resident proteins (PDIA1, PDIA4, PLOD2, HYOU1, CALR) and all decreased at 25°C. Other stress response proteins that were down-regulated are associated with DNA replication/repair (PCNA), transcriptional repression (CSDA), pre-mRNA processing (NONO) and regulation of translation (PABPC4). There was, however, up-regulation of the non-histone chromatin-binding protein HMGB1, the anti-inflammatory mediator annexin A1 (ANXA1), the adapter protein 14-3-3 zeta and 3 proteins associated with cell adhesion (FN1, TSP1, CTGF).

### Cytoskeletal proteins

**Figure 7:**
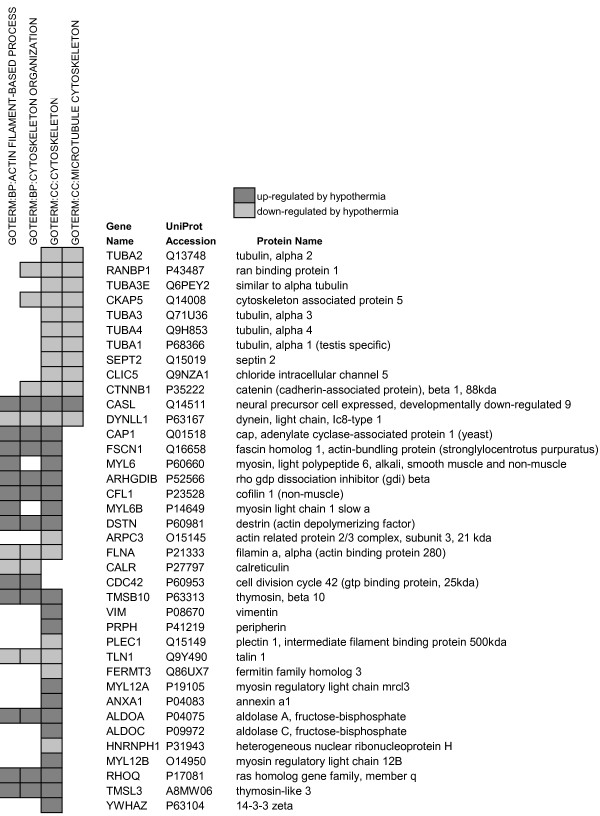
**Cytoskeletal proteins that were significantly up- or down-regulated at 25°C**.

The proteomic findings indicate that there were significant changes in the cytoskeletal organization of cold-adapted cells, including changes in the abundance of 36 proteins associated with microtubule, actin filament and intermediate filament networks (Figure 7). Eleven out of 12 identified proteins of the microtubule cytoskeleton decreased significantly, including α-tubulins α1, α2, α3, α3E, and α4, as well as proteins involved in microtubule assembly (RANBP1), transport (CKAP5) and stability (CKAP2). The decrease in α-tubulin abundance was matched by a decrease in chaperonins CCT4 and CCT5 (Figure 6), which assist in actin and tubulin folding.

Thirteen out of 17 proteins associated with actin filament (F-actin)-based processes were up-regulated in cold-adapted cells (Figure 7). Although there were no significant changes in the abundance of cytosolic actin at 25°C (see Additional file 1), there were significant decreases in actin nucleation and cross-linking proteins (ARPC3 [43] and FLNA [44]), increases in actin filament severing proteins (CFL1 and DSTN [45]) and in proteins that inhibit polymerization by binding to monomeric G-actin (CAP1 [46], TMSB10 [47] and TMSL3). There was also a decrease in septin 2, a GTP-binding and actin-organizing protein [48]. Cold-adaptation increased several other proteins that associate with actin, including the membrane-associated small GTPase RHOQ; Rho GDP dissociation inhibitor β (ARHGDIB), a chaperone protein that inactivates Rho GTPases [49]; CDC42, a Rho GTPase that regulates actin cytoskeletal remodeling; the actin-bundling protein fascin (FSCN1); and myosin light chain subunits (MYL6, MYL6B, MYL12A and MYL12B), which regulate actinomyosin contractility.

Other changes included a cold-induced increase in the type III intermediate filament (IF) proteins vimentin (VIM) and peripherin (PRPH) that form a third class of cytoskeletal network (Figure 7). There were changes in the abundance of proteins (CASL, TLN1, PLEC1, and FERMT3) that link the cytoskeleton to integrin, which is a membrane receptor that binds the cell to proteins of the extracellular matrix, and there was a significant decrease in β-catenin (CTNNB1), a cytoplasmic component of the adherens cell-cell junction.

## Discussion

### Oxidative stress and protein oxidation/reduction

Differential proteomic analyses of endothelial cells collected before or after cold-adaptation at 25°C demonstrated significant changes in the abundance of proteins that regulate redox homeostasis or have oxidoreductase activity. The increase in thioredoxin and thioredoxin reductase correlated with the increase in their respective activities and with the recovery of reduced protein thiols following rewarming from 0°C, conditions that generate oxidative stress and endothelial cell damage [16,50,51]. Protein disulfide isomerases catalyze thiol oxidation; therefore, the decrease in PDIA1 and PDIA4 abundance in cold-adapted cells may have contributed to the recovery of PSH with rewarming. Additional adaptations are potentially beneficial to the maintenance of redox homeostasis. For example, preliminary studies showed that there is an increase in tyrosine hydroxylase, the rate-limiting enzyme in catecholamine synthesis, at 25°C [52]. Catecholamines such as dopamine attenuate hypothermia-induced reactive oxygen species and the loss of -SH reducing equivalents at 4°C in human umbilical vein endothelial cells [53].

Endothelial cold-adaptation at 25°C is accompanied by a significant increase in intracellular GSH [16]. The two enzymes required for GSH synthesis, gamma glutamylcysteine synthetase and glutathione synthetase, were not identified in our proteomic study. However, there was a significant increase in S-adenosylhomocysteine hydrolase (SAHH; Figure 2), a component of the methionine-cysteine transsulfuration pathway that generates cysteine for GSH synthesis. This pathway is up-regulated in response to oxidative stress [54] and accounts for up to 50% of GSH production in some tissues [32]. We did not observe an increase in GSSG/tGSH, which is indicative of oxidative stress, with cold-adaptation, but this does not rule out the possibility of localized changes in GSSG/tGSH that could have had a stimulatory effect on tGSH synthesis at 25°C. The increase in protein glutathionylation following rewarming from 0°C matched the higher levels of tGSH in cold-adapted cells [16] and the increase in abundance and activity of glutathione S-transferase. GST conjugates GSH to proteins under conditions of oxidative stress [55]. Protein glutathionylation is a reversible process that precludes irreversible disulfide cross-linking [56]; therefore, glutathionylation is potentially a protective mechanism in cold-adapted cells.

### NAD metabolic proteins

The increase in GSH/GST and Trx/TrxR activities requires the availability of reducing equivalents from NADPH. Cold-adapted cells had a significant increase in Nampt, the rate-limiting enzyme in NAD synthesis from nicotinamide [30]. NAD kinase, which phosphorylates NAD^+^(H) to NADP^+^(H), was not identified in our study. The enzymes of the pentose phosphate pathway (PPP) that reduce NADP^+ ^to NADPH, glucose-6-phosphate dehydrogenase and 6-phosphogluconate dehydrogenase, were unchanged in abundance. However, the decrease in phosphofructokinase (PFK), the rate-limiting enzyme in the glycolytic pathway, potentially redirects glucose catabolism from glycolysis to the alternative PPP in a manner similarly induced by down-regulating the activity of other downstream glycolytic enzymes such as GAPDH or triosephosphate isomerase (TPI) [57]. The proteomic data indicate that there may be two additional mechanisms in cold-adapted cells to bolster NADPH availability. Aldehyde dehydrogenase (ALDH1A1- Figure 2), which oxidizes the lipid peroxidation products 4-hydroxynonenal and malondialdehyde using NADP^+ ^as a cofactor [34], increased at 25°C. Lipid peroxidation is an important mechanism of injury during 0°C storage and rewarming [50,51]. An increase in ALDH1A1 would therefore potentially protect cell membranes from oxidative damage and simultaneously regenerate NADPH for GSSG and Trx reduction. A second approach to ensuring NADPH availability relates to the apparent decrease in NADPH-dependent biosyntheses at 25°C. Ribonucleotide reductase (RRM1), a Trx-dependent enzyme [35] that catalyzes deoxyribonucleotide formation and controls the rate of DNA synthesis at 37°C [36], decreased significantly at 25°C (Figure 2). Inosine monophosphate dehydrogenase (IMPDH2), the rate limiting enzyme in *de novo *guanine nucleotide synthesis, also decreased at 25°C. Inhibition of IMPDH2 decreases guanine nucleotide pools and interrupts both DNA and RNA synthesis [58]. Down-regulating these two enzymes may therefore suppress DNA synthesis independently of the Q_10 _effects [59] and increase the availability of NADPH for GSSG and Trx reduction. A significant decrease in the NADPH-dependent fatty acid synthase (Figure 2), and presumably fatty acid synthesis, at 25°C may have a similar NADPH-sparing effect and thereby contribute to the protective phenotype of cold-adapted cells.

### Glycolysis

**Figure 8:**
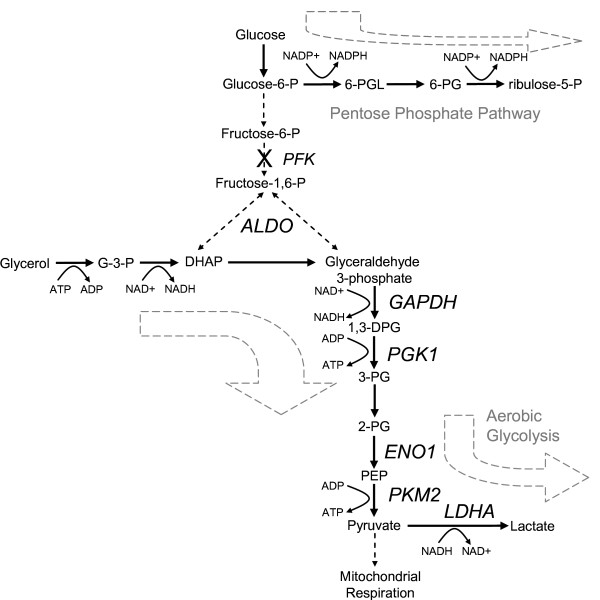
**Hypothetical changes in the energy metabolism of cold-adapted endothelial cells**. The rate-limiting enzyme, phosphofructokinase (PFK), was down-regulated at 25°C and potentially redirects the carbohydrate flux into the pentose phosphate pathway for increased reduction of NADP^+ ^to NADPH. Aldolase (ALDOA, ALDOC), glyceraldehyde phosphate dehydrogenase (GAPDH), phosphoglycerate kinase (PGK1), enolase (ENO1, ENO2, ENO3), pyruvate kinase (PKM2). were up-regulated at 25°C, implicating a possible inflow of intermediate metabolites, like glycerol, into the glycolytic pathway. The increase in lactate dehydrogenase (LDHA, LDHAL6A, LDHAL6B) suggests that cold-adapted endothelium may have a greater reliance on aerobic glycolysis and a lesser reliance on mitochondrial respiration for their energy requirements. 6-PGL, 6-phosphoglucono-δ-lactone; 6-PG, 6-phosphogluconate; G-3-P, glycerol-3-phosphate; DHAP, dihydroxyacetone phosphate; 1,3-DPG, 1,3-diphosphoglycerate; 3-PG, 3-phosphoglycerate; 2-PG, 2-phosphoglycerate; PEP, phosphoenolpyruvate.

*P-values *and *fold-enrichment *values derived from the bioinformatic analysis (Table 2) indicate that one of the most important adaptations at 25°C may be changes to glycolysis. There are 10 steps in the conversion of glucose to pyruvate [60] and the enzymes that catalyze 6 of the final 8 steps changed significantly in abundance with cold-adaptation (Figure 8). Contrary to the aforementioned decrease in PFK, however, there was an increase in the abundance of enzymes that catalyze 5 of the remaining 7 steps in glycolysis downstream of PFK, including aldolase, glyceraldehyde phosphate dehydrogenase, phosphoglycerate kinase, enolase, and pyruvate kinase (ALDOA/ALDOC, GAPDH, PGK1, ENO1/ENO2/ENO3 and PKM2; Figure 2). This inconsistency is reconcilable if there is an increase in intermediate metabolites entering the glycolytic pathway. For example, glycerol is produced by the breakdown of triglycerides to fatty acids and is converted into glycerol 3-phosphate and then into dihydroxyacetone phosphate (DHAP), a glycolytic intermediate. The conversion of glycerol to DHAP and further oxidation to pyruvate would yield one mole of ATP per mole of glycerol consumed compared to two moles of ATP per mole of glucose. Glycerol is a substrate for gluconeogenesis under some conditions [61] but it has also been identified as an important energy substrate in the ex vivo rodent heart [62,63], cultured cardiomyocytes [64] and the rodent brain [65]. A role for glycerol in endothelial energy metabolism during adverse conditions has not been reported.

Cold-adapted cells had a significant increase in lactate dehydrogenase (LDHA, LDHAL6A and LDHAL6B), which reduces pyruvate to lactate. This step diverts pyruvate from further oxidation by mitochondrial respiration and regenerates NAD^+ ^to facilitate more glycolysis. The reduction of pyruvate despite the availability of oxygen is known as the Warburg effect or *aerobic glycolysis *and has been shown to decrease mitochondrial respiration, mitochondria-derived ROS and apoptosis [66,67]. In summary, the observed changes in the abundance of glycolytic proteins may be a cold-induced adaptation that promotes NADPH reduction while maintaining ATP generation and minimizing mitochondria-generated oxidative stress.

### Proteins that regulate gene expression

Cold-adaptation induced significant changes in the abundance of proteins that regulate gene expression. Histones and histone modifications, such as methylation or acetylation, play an important role in regulating transcription by modifying chromatin structure [68]. The histone H2A.V increased significantly at 25°C. In *Arabidopsis *and in budding yeast cells, incorporation of the closely related variant H2A.Z [69] into nucleosomes is a requirement for direct temperature-induced changes in gene expression [70]. Whether or not H2A.V plays a similar role in promoting the expression of cold-responsive endothelial genes is unknown.

Histone methylation mediates short-term chromatin condensation and transcriptional repression (*DNA methylation *affects the long-term silencing of genes) [71]. In addition to its role in generating cysteine for GSH synthesis, S-adenosylhomocysteine hydrolase (SAHH) is critical to histone/DNA methylation and gene silencing because it eliminates S-adenosylhomocysteine (SAH), a by-product and potent inhibitor of S-adenosylmethionine (SAM)-dependent methyltransferase reactions [72] (SAM is the methyl donor). The increase in SAHH observed at 25°C may therefore promote histone/DNA methylation and potentially gene silencing [73]. There is also experimental evidence that increases in Nampt, which we observed at 25°C, promotes histone deacetylation and consequently gene silencing [74]. The level of NAD and Nampt in mouse fibroblasts was shown to regulate the activity of Sir2, a protein deacetylase [31]. Similarly, the overexpression of Nampt in smooth muscle cells enhanced cell resistance to oxidative stress and increased the activity of SIRT1, the human homolog of Sir2 [75]. SIRT1 regulates nucleosome and chromatin structure by deacetylating histones H3 and H4 and mediates stress resistance, apoptosis and inflammatory responses by deacetylating the transcription factors FOXO, p53 and NF-κB [76]. The increase in the expression of SAHH and Nampt at 25°C, and their capacities to modulate both transcriptional repression and resistance to oxidative stress, suggests that these proteins may have pivotal roles in the adaptation to cold.

Additional findings point to an overall decrease in transcription and translation at 25°C. There were significant decreases in the abundance of proteins associated with transcription initiation, mRNA elongation, processing and nonsense-mediated decay, and stress granule assembly. The inhibition of IMP dehydrogenase has been shown to disrupt DNA and RNA synthesis [42] and its decrease at 25°C potentially limits global transcription and translation. The increase in transcriptional repressors ENO1 and HNRNPDL at 25°C complement earlier findings that showed increases in the repressors prohibitin, L3MBTL2 and Zinc finger protein 224 [52]. Although ENO1 (α-enolase) is recognized as a glycolytic protein, an alternate translation product of the ENO1 mRNA is a repressor of the c-myc gene [77]. The myc family of proteins are transcription factors that regulate ribosome biogenesis, protein synthesis, cell growth and proliferation [78]. Although we did not identify c-myc, N-myc was identified and it had the largest fold-change decrease (-2.38) of any protein detected in this study (Additional file 1; N-myc was excluded from the analysis because of its classification as a priority 4 protein [24,26]). However, the decrease in N-myc abundance and increase in ENO1 match the apparent decrease in ribonucleoprotein complex biogenesis (11 out of 11 proteins decreased), translation (38 out of 40 decreased) and unfolded protein binding (9 out of 9 decreased). By contrast, cold-adaptation increased the abundance of RBM3, a cold-inducible RNA-binding protein that increases the efficiency of global protein synthesis at 37°C and at mild hypothermic temperatures [42]. It has been suggested that RBM3 may prevent a disproportionate cold-induced decrease in translation [42]. The fact that one third of the changes in protein abundance at 25°C were *increases *suggests that RBM3 may have an important role in facilitating these increases.

### Response to stress proteins

The *response to stress *proteins (Figure 6) that increased or decreased significantly with cold-adaptation are associated with processes like chromatin binding, transcription, DNA repair, pre-mRNA processing, regulation of translation, protein folding and cell adhesion. Five of the identified proteins are found in the ER, which is the site where secretory and cell surface proteins are synthesized, folded and modified [79]. The decrease in the abundance of these ER-resident proteins after 72 h at 25°C is additional evidence that protein synthesis and protein folding is repressed in the cold-adapted state. Excess unfolded or misfolded proteins in the ER activates the Unfolded Protein Response or *UPR*. The UPR initiates translational repression, increases the transcription of ER chaperone genes and increases the degradation of misfolded proteins to re-establish the equilibrium between protein synthesis and protein folding reactions [79]. It is not known if ER function is impaired at 25°C or if translational repression due to hypothermia is initiated by the UPR, but evidence of UPR activation in the tissues of hibernating mammals has been reported [80]. Activation of the UPR is also a potential mechanism for increasing GSH in cold-adapted cells [16] because of the important role that GSH plays in regulating redox homeostasis during the oxidative folding of proteins [29]. ER stress-activated transcription factors ATF4 and Nrf2 increase GSH synthesis [81,82] and increases in endothelial GSH have been linked to hyperoxic- or cycloheximide-induced suppression of translation [83].

YB-1 and CSDA are two nucleic acid-binding stress proteins that contain a central *cold-shock domain*, a 100-amino acid sequence that derives its name from its resemblance to bacterial cold-shock proteins [38]. In homeothermic organisms, cold-shock domain (CSD) proteins have been associated with the transcriptional regulation of stress-response, growth factor, and proliferation-associated genes [38]. In bacteria, cold-shock proteins function as mRNA chaperones that facilitate translation [84]. Their expression levels increase during the acclimation to cold and then later decline below pre-cold-shock levels [85]. It is this archaic function of CSD proteins that may have special relevance to the endothelial adaptation to cold.

Cold-adapted cells had significantly higher levels of 14-3-3 proteins zeta/delta (Figure 6, 7) and theta (Figure 5). 14-3-3's are a family of conserved proteins that control many diverse processes by binding to specific phosphorylated sites on target proteins to induce changes in the target conformation or to change the target's interactions with other proteins [86]. In plants, 14-3-3's help regulate processes such as photosynthesis, ATP production and nitrate reduction in response to changing environmental factors including *cold stress*[87]. In mammalian cells, 14-3-3's regulate responses to growth factors, including the stimulation of glycolysis, inhibition of apoptosis and inhibition of transcription [86].

### Cytoskeletal proteins

The adaptation of endothelial cells to cold induced significant changes in the abundance of cytoskeletal proteins that implicate diminished microtubular and actin networks, an expanded intermediate filament network and modified cell-cell and cell-extracellular matrix interactions. The actin cytoskeleton underlies the plasma membrane to provide support, resist tension and maintain cell shape. However, it is a highly dynamic structure that responds to environmental stimuli and rapidly reorganizes to change cell shape, form membrane protrusions, (such as lamellipodia and filopodia for cellular locomotion), or form stress fibers (bundles of radially oriented actin-filaments cross-linked with myosin motor proteins) for retracting the plasma membrane. The proteomic findings suggest that there is a significant reorganization of the actin cytoskeleton in response to the reduced temperature. Decreases in actin nucleation and cross-linking proteins (ARPC3 [43] and FLNA [44]), increases in actin filament severing proteins (CFL1 and DSTN [45]) and in proteins that bind to G-actin to inhibit polymerization (CAP1 [46] and TMSB10 [47]), allude to a net disassembly of the actin cytoskeleton at 25°C. Disassembly is further indicated by the increase in RhoGDI2, which promotes actin depolymerization [49], and by the decrease in septin 2, whose depletion leads to dissociation of actin stress-fibers [48].

Microtubules consist of α- and β-tubulin heterodimers and form robust structures that are resistant to bending or compression [88]. Microtubules organize the cytoplasm, position the nucleus and organelles, transport organelles and make up the mitotic spindle [88]. The decrease in α-tubulins, microtubule associated proteins and chaperonins at 25°C indicate a decrease in the microtubule network (11 out of 12 microtubule-associated proteins decreased). Our preliminary studies showed a matching increase in the microtubule stabilizing protein LIS1 at 25°C [52], which points to a decrease in microtubule catastrophe events and diminished microtubule dynamics [89]. One consequence of these changes may be a reduction in GTP utilization because microtubule assembly/disassembly requires the hydrolysis of tubulin-bound GTP to GDP [88]. Such a reduction would match the apparent decrease in translation, which is another GTP-utilizing process, and the decrease in IMPDH2 (Figure 2), which is the rate-limiting enzyme in de novo GMP synthesis.

In contrast to diminished microtubular and actin-filament networks, there was a cold-induced increase in type III intermediate filaments (IFs; Figure 7) that form a third class of cytoskeletal network. Cells at 25°C developed a significant increase in vimentin, a major IF that provides mechanical support for the cell and takes part in additional processes such as the regulation of cell adhesion to the extracellular matrix. A second IF, peripherin, also increased. IFs are highly dynamic structures that self-assemble without expenditure of GTP or ATP required by tubulin or actin polymerization, respectively. It has been theorized that since actin dynamics may account for up to 50% of ATP turnover in resting cells, a change in cytoskeletal structure to a less dynamic form may reflect a decrease in the energy state of the cell or possibly represent a form of ATP preservation for more crucial activities [90]. The substitution of an expanded IF network for diminished actin filament and microtubule networks may therefore be a possible sign that cold-adapted cells are in an energy-reduced or energy-conserved state.

Cytoskeletal changes such as those noted above are frequently mediated through changes in cell-cell and cell-matrix adhesion and our study indicates that similar processes are occurring with cold-adaptation. Cdc42, a Rho GTPase that regulates actin cytoskeletal remodeling in response to environmental signals, increased significantly at 25°C. Activation of Cdc42 preserves the integrity of endothelial cell-cell junctions [91], prevents membrane retraction [91] and induces filopodia formation [92]. These properties may contribute to the improved ability of cold-adapted endothelial cells to reverse intercellular gap formation during rewarming from 0°C [16]. The increase in Cdc42 in our study coincided with significant increases in fascin, a protein that organizes actin filaments into the parallel bundles that are incorporated into filopodial protrusions [93]; in myosin light chain subunits (MYL6, MYL6B, MYL12A and MYL12B) that regulate actinomyosin contractility and filopodia formation [94]; and in fibronectin 1 and thrombospondin 1 (Figure 6), which are secreted components of the extracellular matrix. The association of fascin with actin is regulated by the binding of integrin, a transmembrane receptor, to the extracellular matrix and to thrombospondin and fibronectin in particular [95]. In addition, there were changes in the abundance of proteins that link integrin to the cytoskeleton. For example, CasL, which mediates signals between integrin and possibly vimentin [96], and talin 1, which links integrin to actin [97], increased significantly. By contrast, plectin, a linker protein between IFs and microtubules, microfilaments or integrins [98], and kindlin-3, which is a co-activator of integrin [99], decreased at 25°C. Cold-adaptation also caused changes in components of cell-cell complexes. There were significant decreases in β-catenin, which links the adherens cell-cell junction to F-actin [100]. Our previous findings showed decreases in the membrane receptor EphA3, which is a member of the Eph receptor family of receptor tyrosine kinases that induce changes in cell shape and movement upon the binding of ephrin ligands from an adjacent cell [101]. Overall, these findings suggest that cytoskeletal remodeling during cold-adaptation may be stimulated by increases in fibronectin and thrombospondin secretion, integrin binding and cdc42 activation, and by decreases in adherens junction and ephrin/ephrin receptor cell-cell signaling.

### Lipids/lipid fluidity

Organisms that successfully adapt to cold change the lipid composition of their cell membranes to maintain membrane fluidity and function with decreasing temperature. Some cold-induced membrane changes observed in fish are an increase in the proportion of unsaturated to saturated fatty acids, an increase in fatty acid chain length and an increase in the proportion of phosphatidylethanolamine to phosphatidylcholine [102]. These changes are brought about by adjustments in lipid metabolism, including de novo synthesis, elongation and desaturation of fatty acids; de novo phospholipid synthesis and desaturation; and phospholipid catabolism and turnover in the membrane [102]. In our study, few lipid metabolic proteins were identified so there is little information about potential modifications to endothelial cell membranes in response to cooling. Fatty acid synthase was significantly down regulated in cold-adapted cells (Figure 2), which suggests that de novo fatty acid synthesis is not essential. Interestingly, there was a cold-induced increase in phospholipase A_2 _receptor 1 (PLA2R1 was a priority 4 protein; see Additional file 1), which binds the lipolytic enzyme phospholipase A_2 _(PLA_2_). PLA_2_s hydrolyze phospholipids at the sn-2 position, releasing free fatty acids and lysophospholipids from the membrane. The sn-2 position is frequently occupied by polyunsaturated fatty acids (PUFA), so PLA_2 _activity potentially regulates membrane unsaturation and fluidity. In support of this hypothesis, Zhang et al. demonstrated that inhibition of Ca^2+^-independent PLA_2 _activity increased PUFA in membrane phosphatidylcholines [103]. PLA_2 _activity at hypothermic temperatures is well-known [104-106]. It is not known, however, if PLA2R1 has a stimulatory or inhibitory effect on endothelial PLA_2_s.

In addition to a direct effect of cooling on membrane fluidity, there may be an indirect effect that is caused by cold-induced oxidative stress and peroxidation of unsaturated lipids. The lipid peroxidation products 4-hydroxynonenal and malondialdehyde have been shown to increase the rigidity of cell membranes [107,108] and have been measured following cold exposure and rewarming [50,51]. The increase in peroxiredoxin, aldehyde dehydrogenase and glutathione S-transferase in cold-adapted cells potentially attenuate lipid peroxidation in endothelial membranes and may help preserve membrane fluidity during cooling.

### Comparisons to earlier proteomic studies

This study was performed using a well-validated label-free mass spectrometry-based method [24,26,109,110] that is rapid, sensitive and has a three- to four-fold higher protein dynamic range (protein mass and abundance) than two-dimensional gel electrophoresis (2-DE) [111]. Almost one third of the 1089 proteins that were identified significantly increased or decreased in abundance during cold-adaptation and 181 proteins of high identification confidence were included in the functional analysis. This number greatly exceeds the number of proteins identified and analyzed in previous 2-DE-based hypothermia studies [112,113]. Ten of 26 proteins identified by Baik et al. [112] changed significantly in our study, including the glycolytic proteins pyruvate kinase M2, phosphoglycerate kinase and glyceraldehyde-3-phosphate dehydrogenase; cytoskeletal proteins α-tubulin, annexin A1 and vimentin; chaperones HSP90β and HSPA8; and the ER-resident protein disulfide isomerase. With the exception of PDI and HSPA8, the changes in abundance were in the same direction as in our study. Kumar et al. [113] observed similar cold-induced increases in the abundance of vimentin, aldehyde dehydrogenase 1A1 and GAPDH as were reported in this study. They also reported decreases in tubulins (β-tubulin rather than α-tubulin) and annexins (A4 rather than A1) [113]. In addition, Kumar et al. [113] identified a decrease in α-enolase (we noted an increase) and an increase in the cytoskeletal chaperone CCT2 (we reported decreases in CCT4 and 5).

## Conclusions

**Figure 9:**
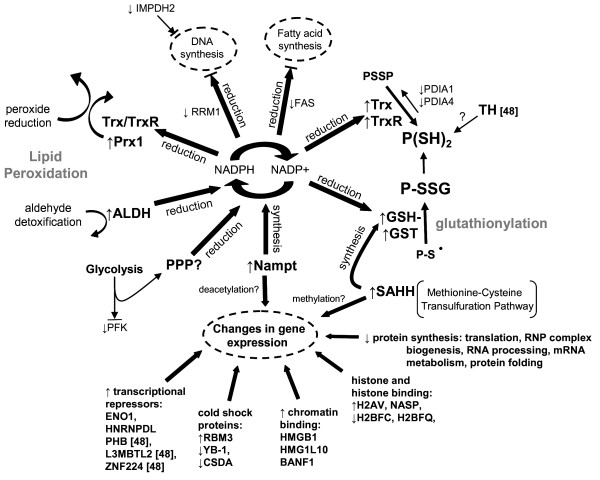
**Endothelial adaptive response to moderate hypothermia (25°C/72 h)**. See *Conclusions *for details. Thioredoxin (Trx), thioredoxin reductase (TrxR), tyrosine hydroxylase (TH), reduced glutathione (GSH), glutathione S-transferase (GST), protein disulfide isomerase (PDIA1 and 4), reduced protein thiols (P(SH)_2_), protein disulfide (PSSP), glutathionylated protein (P-SSG), oxidized protein (P-S•), S-adenosylhomocysteine hydrolase (SAHH), peroxiredoxin 1 (Prx1), aldehyde dehydrogenase (ALDH), nicotinamide phosphoribosyltransferase (Nampt), reduced/oxidized nicotinamide adenine dinucleotide phosphate (NADPH/NADP^+^), fatty acid synthase (FAS), ribonucleotide reductase M1 (RRM1), inosine-5'-monophosphate dehydrogenase 2 (IMPDH2), phosphofructokinase (PFK), pentose phosphate pathway (PPP), histone (H2AV, H2BFC, H2BFQ), histone binding proteins (NASP or nuclear autoantigenic sperm protein), chromatin binding proteins (High-mobility group protein B1 (HMGB1), high-mobility group protein 1-like10 (HMG1L10), barrier to autointegration factor 1 (BANF1)), RNA binding motif protein 3 (RBM3), Y-box binding protein 1 (YB-1), cold-shock domain protein A (CSDA), prohibitin (PHB), enolase 1 (ENO1), lethal(3)malignant brain tumor-like 2 protein (L3MBTL2), heterogeneous nuclear ribonucleoprotein D-like (HNRNPDL), Zinc finger protein 224 (ZNF224).

Differential proteomic analysis of endothelial cells collected before or after cold-adaptation at 25°C demonstrated significant changes to a broad range of biological processes and a new phenotype of enhanced redox protection and down-regulated anabolic and energy-consuming processes. The principal findings are depicted in Figure 9 and provide a preliminary model of the endothelial adaptive response to cold and a framework to guide future investigations. Cold-adaptation increases the abundance of redox active proteins thioredoxin, thioredoxin reductase, glutathione S-transferase and tyrosine hydroxylase [52] and decreases protein disulfide isomerases 1 and 4 and glutaredoxin-3 [52]. These changes correlate with a recovery of protein thiols, an increase in protein glutathionylation and diminished oxidative stress when cells are rewarmed from 0°C. Protein glutathionylation may prevent irreversible oxidation and formation of intermolecular disulfide bonds and therefore represents a potentially important protective function of GSH in cold-adapted cells. The increase in abundance of S-adenosylhomocysteine hydrolase (SAHH), a component of the methionine-cysteine transsulfuration pathway that generates cysteine for GSH synthesis, may play a role in generating the higher concentrations of GSH observed in cold-adapted cells [16]. In addition to protecting proteins from oxidative injury, cold-adapted cells may be more resistant to lipid peroxidation than non-adapted cells because of increases in peroxiredoxin 1 and aldehyde dehydrogenase (ALDH), which enzymatically reduce lipid peroxides and detoxify aldehydes, respectively. The increase in antioxidant activities requires the availability of reducing equivalents from NADPH and the proteomic data show several adaptive changes that could increase NADPH availability. Cold-adapted cells have higher levels of Nampt, the rate-limiting enzyme in NAD synthesis from nicotinamide. A significant decrease in PFK, the rate-limiting enzyme of glycolysis, potentially redirects glucose catabolism to the pentose phosphate pathway to increase NADP^+ ^reduction to NADPH. The increase in ALDH abundance (and presumably activity) also potentially increases NADP^+ ^reduction. Most importantly, the attenuation of NADPH-dependent anabolic processes such as DNA, RNA and fatty acid synthesis due to decreases in RRM1, IMPDH2, and FAS expression, respectively, could further increase NADPH availability for antioxidant activities.

Central to the proteomic changes is the evidence of a regulated decrease in energy consuming processes such as protein synthesis, DNA synthesis, fatty acid synthesis and cytoskeletal dynamics. The net decrease in proteins associated with translation, ribonucleoprotein complex biogenesis, RNA processing, mRNA metabolism and protein folding at 25°C is consistent with a generalized cold-induced inhibition of protein synthesis described by others [19]. Increases in transcriptional repressors and changes in the abundance of histone and non-histone chromatin-binding proteins are possible indicators of a concurrent decrease in transcription. Changes in the expression of cold-shock proteins (RBM3 increased, CSDA and YB-1 decreased) and the increase in histone H2A.V at 25°C potentially counteract the generalized decrease in gene expression by enhancing translation efficiency and the direct responsiveness of genes to cold, respectively. This may be an important requirement for increasing the synthesis of a subset of proteins at reduced temperatures- one third of identified proteins *increased *in abundance in our study. Two enzymes we have identified, SAHH and Nampt, may play pivotal roles in mediating adaptation to cold because each induces transcriptional repression and increases the cell's reducing capacity. SAHH facilitates SAM-dependent DNA and histone methylation and consequently gene silencing [73] while up-regulating GSH synthesis. Nampt regulates histone deacetylation and transcriptional repression [74] while increasing NAD synthesis. The ability to down-regulate cellular energy demand while increasing the cell's reducing capacity would be clinically beneficial where the application of cold-ischemia would otherwise create energy insufficiency, uncouple metabolic integration and generate oxidative stress and injury. Whether or not a similar adaptive response occurs in other cell types, such as myocardial or neural cells, or following an ischemic insult, such as during the application of therapeutic hypothermia, remain important questions.

## Methods

### Cell culture

Proliferating human coronary artery endothelial cells (Clonetics; passages 4 to 8) were seeded at a density of 1.4 × 10^4 ^viable cells/cm^2 ^(6-well culture plates, 12.5 cm^2^, 25 cm^2 ^or 75 cm^2 ^culture flasks; Falcon, Franklin Lakes, New Jersey) and were maintained at 37°C (95% air + 5% CO_2_) in culture medium (EGM2-MV; Clonetics). Cells formed a confluent monolayer after 3 to 4 days of culture and were then used in experiments. Cells were adapted to cold at 25°C for up to 72 h in a water-jacketed incubator (3.8% CO_2 _+ 96.2% air) and the culture medium was replaced with fresh medium every 24 h. Controls consisted of confluent cells collected at the start of the adaptation period or following continued maintenance at 37°C for up to 72 h. For cold storage, cells were washed with Hank's balanced salt solution, flasks received endothelial basal medium (EBM-2, Clonetics) and were immersed in a circulating water bath at 0°C.

### Label-free protein quantification method

A large scale quantitative proteomics experiment was performed using a well-validated label-free mass spectrometry-based method [24,26,109,110]. Cold-adapted and control cells were prepared as previously described [110]. Proteins were extracted from the tissue culture cells using 8 M urea, reduced by triethylphosphine, alkylated by iodoethanol, and digested by trypsin [114]. Tryptic peptides (~20 μg/injection) were analyzed using Thermo-Fisher linear ion-trap mass spectrometer (LTQ) coupled with a Surveyor HPLC system. C-18 reverse phase column (i.d. = 2.1 mm, length = 50 mm) was used to separate peptides with a flow rate of 200 μL/min. Peptides were eluted with a gradient from 5 to 45% acetonitrile developed over 120 min and data were collected in the triple-play mode (MS scan, zoom scan, and MS/MS scan). The acquired data were filtered and analyzed by a proprietary algorithm that was developed and described by Higgs, et al. [26,109]. Database searches against the IPI (International Protein Index) human database was carried out using both the X!Tandem and SEQUEST algorithms. Protein quantification was also carried out using the same proprietary algorithm described previously [24,26,110].

### Bioinformatics analysis

IPIs of Priority 1 and 2 proteins were converted to UniProt Accession numbers using the EMBL-EBI PICR Service, http://www.ebi.ac.uk/Tools/picr/. Accession numbers were then uploaded to the DAVID (Database for Annotation, Visualization and Integrated Discovery) Bioinformatics Resources 6.7 website http://david.abcc.ncifcrf.gov/tools.jsp and the proteins were classified and organized according to their GO *Biological Process, Molecular Function, Cell Component *and *SP-PIR Keyword *annotations using the Gene Functional Annotation Tool.

### Measurement of protein thiols (PSH) and mixed disulfides (PSSG)

Confluent HCAEC from T-75 flasks were rinsed twice with HBSS and scraped in 1.5 ml of 5% TCA in 10 mM HCl with 5 mM EDTA. Protein pellets and non-protein supernatants were separated by centrifugation and stored at -80°C until analysis of protein thiols (PSH) and mixed disulfides (PSSG) using the method described by Nagy [115] with some modifications. The protein pellets were extracted with diethyl ether to eliminate TCA, redissolved in 0.25 ml 6 M GuHCl, and pH adjusted to 8 by addition of 0.5 ml phosphate buffer with 5 mM EDTA, pH 8.6. Insoluble material was removed by centrifugation and supernatants were used to determine the levels of PSH and PSSG. For PSH, 100 μl of the sample was reacted with 0.9 ml of 0.1 mM 5,5'-dithio-bis-(2-nitrobenzoic acid) (DTNB, 1 mM stock in methanol) in 125 mM sodium phosphate buffer containing 6.25 mM EDTA, pH 8.6. Absorbance was measured at 412 nm after 30 minutes at room temperature. GSH standards (50-500 μM) were similarly measured and PSH-levels in samples were calculated directly from the standard curve.

PSSG levels were determined in the reaction mix comprised of 0.1 ml sample, 0.9 ml of 125 mM sodium phosphate buffer, pH 7.5 (with 6.25 mM EDTA) containing 0.1 mM DTNB, 0.03 mM NADPH and 20 μl of glutathione reductase (25 units per ml). The absorbance was measured at 412 nm after 10 minutes of incubation at room temperature. PSSG levels in samples were calculated from a GSH standard curve (0.5 - 10 μM) run under same conditions.

### Glutathione determination

Confluent cells were washed two times in HBSS and then lysed in 500 μL of 125 mM sodium phosphate buffer with 6.3 mM EDTA and 2% Triton X-100. Protein was removed from lysates by precipitation with 500 μL of metaphosphoric acid (10% w/v) and centrifugation at 14,000 × *g *for 5 minutes. The supernatant was neutralized with 5 N sodium hydroxide and stored at -80°C until t-GSH and GSSG was measured using the method of Ishii [116]. To determine t-GSH, 100 μL of the neutralized supernatant was incubated at 25°C with 800 μL of 0.3 mM NADPH in 125 mM sodium phosphate buffer with 6.3 mM EDTA, 100 μL of 6 mM 5,5'-dithiobis(2-nitrobenzoic acid) (DTNB) and 20 μL of 25 U/mL GSH reductase. After precisely 15 minutes, the optical density was measured at 412 nm. To determine GSSG, GSH in samples was derivatized by adding 2.5 μL of 4-vinylpyridine (95%) per 250 μL solution, mixing vigorously for 1 min and incubating samples at room temperature for 30 min. Samples were then centrifuged at 14,000 × *g *for 5 min and GSSG was measured in supernatants by the procedure described for t-GSH. The measured optical densities were converted to concentrations using standard curves prepared with GSH (0.5-10 μM) or GSSG (0.25-5 μM).

### Measurements of thioredoxin, thioredoxin reductase, and glutathione S-transferase activity

Confluent HCAECs from T-75 flasks were scraped and resuspended in 0.15-0.5 ml of assay buffer and cell lysates were prepared by sonicating samples two times on ice (10 seconds, amplitude of 40) using an Ultrasonic Processor. Insoluble materials were removed by centrifugation at 13,000 rpm for 20 minutes at 4°C and supernatants were used to measure enzyme activities and total protein concentrations (Micro Bicinchoninic Assay Kit; Pierce Thermoscientific). Thioredoxin (Trx) activity in samples was determined using an assay kit (Redoxica, Littlerock, AK) and following the manufacturer's recommendations. Briefly, Trx activity was determined by measuring the rate of depletion of NADPH at 340 nm in the presence of oxidized insulin and thioredoxin reductase (TrxR). Reaction blanks with each sample were run without the insulin substrate to determine Trx-independent NADPH depletion. Positive controls consisting of thioredoxin supplied with the kit were run to validate the assay. Reaction rates were calculated from the slopes of logarithmic plots of A_340 nm _versus time (in min). The difference in reaction rates between the samples and corresponding reaction blanks represented Trx activity and was expressed as nmoles of Trx activity/min/mg protein.

TrxR activity was measured using an assay kit (Cayman Chemical Company, Ann Arbor, Michigan, USA). The assay is based on measuring the rate of reduction of DTNB (5,5'-dithiobis(2 dinitrobenzoic acid)) to a colored product by TrxR, in the presence of NADPH. TrxR-independent reduction of DTNB was determined by adding aurothiomalate (ATM), a specific inhibitor of TrxR, to the reaction mixture. TrxR activity was determined by measuring the difference in the rate of increase in absorbance at 412 nm between samples with or without ATM and was expressed in nmoles/min/mg proteins, using an extinction coefficient of 6.35 mM^-1^.

GST activities were assessed using an assay kit from Cayman and following the manufacturer's protocol. GST activity was quantified by measuring the rate of conjugation of reduced glutathione by GST to CDNB (1-chloro-2,4 dinitrobenzene) at 340 nm and was expressed in nmoles of conjugated CDNB/min/mg protein, using an extinction coefficient of 0.0053 μM^-1^.

## Abbreviations

IPI: International Protein Index; UniProt: Universal Protein Resource; EMBL: European Molecular Biology Laboratory; EBI: European Bioinformatics Institute; PICR: Protein Identifier Cross Reference; DAVID: Database for Annotation, Visualization and Integrated Discovery; GO: Gene Ontology; SP-PIR: Swiss Prot- Protein Information Resource.

## Authors' contributions

MAJZ conceived of and designed the study, performed cell culture, data analysis and interpretation, bioinformatic analysis, and wrote the manuscript. MPG performed cell culture; measurements of protein thiols, mixed disulfides, oxidized glutathione, and total glutathione; measurements of thioredoxin, thioredoxin reductase and glutathione S-transferase activity; and contributed to data analysis and interpretation. MW contributed to the design of the proteomic study and performed the label-free protein quantification. All authors reviewed and approved of the final manuscript.

## Supplementary Material

Additional file 1 **Summary of identified proteins**. Each protein is listed with its Rank, significant change between control and cold-adapted cells (Signif; Yes or No), Priority, Protein_ID, Protein Name, fold-change (Fold; positive represents an increase in cold-adapted cells; negative represents a decrease), % ID confidence of the best sequence (%ID), qValue (comparing control to hypothermia-treated), mean of control (mean_C; n = 5), mean of cold-adapted (mean_H; n = 5), % Coefficient of Variation (%CV; standard deviation/mean), number of peptides quantified (Peptide#) and peptide sequence with highest ID confidence (Best_Sequence). A detailed description of the parameters are in [24]. Click here for file

Additional file 2 **Summary of biological process, molecular function and cell component categories that changed significantly with cold-adaptation**. Each GOTERM or SP_PIR_KEYWORD is listed with the protein count, percentage of the total (178 proteins), P-value, gene identifiers, number of genes in the category, total number of genes and fold-enrichment. Click here for file

## References

[B1] Apostolakis E Akinosoglou K The methodologies of hypothermic circulatory arrest and of antegrade and retrograde cerebral perfusion for aortic arch surgery Ann Thorac Cardiovasc Surg 2008 14 3 138 148 18577891

[B2] Immer FF Lippeck C Barmettler H Berdat PA Eckstein FS Kipfer B Saner H Schmidli J Carrel TP Improvement of quality of life after surgery on the thoracic aorta: effect of antegrade cerebral perfusion and short duration of deep hypothermic circulatory arrest Circulation 2004 110 11 Suppl 1 II250 255 10.1161/01.CIR.0000138387.61103.a0 15364871

[B3] Thome C Schubert GA Schilling L Hypothermia as a neuroprotective strategy in subarachnoid hemorrhage: a pathophysiological review focusing on the acute phase Neurol Res 2005 27 3 229 237 10.1179/016164105X25252 15845206

[B4] Hildebrand F Giannoudis PV van Griensven M Chawda M Pape H-C Pathophysiologic changes and effects of hypothermia on outcome in elective surgery and trauma patients Am J Surg 2004 187 3 363 371 10.1016/j.amjsurg.2003.12.016 15006564

[B5] Koreny M Sterz F Uray T Schreiber W Holzer M Laggner A Herkner H Effect of cooling after human cardiac arrest on myocardial infarct size Resuscitation 2009 80 1 56 60 10.1016/j.resuscitation.2008.08.019 18951682

[B6] Wolfrum S Pierau C Radke PW Schunkert H Kurowski V Mild therapeutic hypothermia in patients after out-of-hospital cardiac arrest due to acute ST-segment elevation myocardial infarction undergoing immediate percutaneous coronary intervention.[see comment] Crit Care Med 2008 36 6 1780 1786 10.1097/CCM.0b013e31817437ca 18496378

[B7] Bernard SA Buist M Induced hypothermia in critical care medicine: a review.[see comment] Crit Care Med 2003 31 7 2041 2051 10.1097/01.CCM.0000069731.18472.61 12847402

[B8] McIntyre LA Fergusson DA Hebert PC Moher D Hutchison JS Prolonged therapeutic hypothermia after traumatic brain injury in adults: a systematic review.[see comment] Jama 2003 289 22 2992 2999 10.1001/jama.289.22.2992 12799408

[B9] Hidalgo MA Sarathchandra P Fryer PR Fuller BJ Green CJ Effects of hypothermic storage on the vascular endothelium: a scanning electron microscope study of morphological change in human vein J Cardiovasc Surg (Torino) 1995 36 6 525 532 8632019

[B10] Brinkkoetter P-T Beck GC Gottmann U Loesel R Schnetzke U Rudic B Hanusch C Rafat N Liu Z Weiss C Leuvinik HGD Ploeg R Braun C Schnuelle P van der Woude FJ Yard BA Hypothermia-induced loss of endothelial barrier function is restored after dopamine pretreatment: role of p42/p44 activation Transplantation 2006 82 4 534 542 10.1097/01.tp.0000229396.34362.e2 16926598

[B11] Speziale G Ferroni P Ruvolo G Fattouch K Pulcinelli FM Lenti L Gazzaniga PP Marino B Effect of normothermic versus hypothermic cardiopulmonary bypass on cytokine production and platelet function J Cardiovasc Surg (Torino) 2000 41 6 819 827 11232964

[B12] Grunenfelder J Zund G Schoeberlein A Schmid ER Schurr U Frisullo R Maly F Turina M Expression of adhesion molecules and cytokines after coronary artery bypass grafting during normothermic and hypothermic cardiac arrest Eur J Cardiothorac Surg 2000 17 6 723 728 10.1016/s1010-7940(00)00401-2 10856867

[B13] Kevelaitis E Nyborg NC Menasche P Coronary endothelial dysfunction of isolated hearts subjected to prolonged cold storage: patterns and contributing factors J Heart Lung Transplant 1999 18 3 239 247 10.1016/s1053-2498(98)00042-4 10328150

[B14] Redondo J Manso AM Pacheco ME Hernandez L Salaices M Marin J Hypothermic storage of coronary endothelial cells reduces nitric oxide synthase activity and expression Cryobiology 2000 41 4 292 300 10.1006/cryo.2000.2285 11222026

[B15] Wille T de Groot H Rauen U Improvement of the cold storage of blood vessels with a vascular preservation solution. Study in porcine aortic segments J Vasc Surg 2008 47 2 422 431 10.1016/j.jvs.2007.09.048 18164170

[B16] Zieger MAJ Gupta MP Hypothermic preconditioning of endothelial cells attenuates cold-induced injury by a ferritin-dependent process Free Radic Biol Med 2009 46 5 680 691 10.1016/j.freeradbiomed.2008.12.004 19135523

[B17] Glofcheski DJ Borrelli MJ Stafford DM Kruuv J Induction of tolerance to hypothermia and hyperthermia by a common mechanism in mammalian cells J Cell Physiol 1993 156 1 104 111 10.1002/jcp.1041560115 8314851

[B18] Al-Fageeh MB Smales CM Control and regulation of the cellular responses to cold shock: the responses in yeast and mammalian systems Biochem J 2006 397 2 247 259 10.1042/BJ20060166 PMC1513281 16792527

[B19] Sonna LA Fujita J Gaffin SL Lilly CM Invited review: Effects of heat and cold stress on mammalian gene expression J Appl Physiol 2002 92 4 1725 1742 10.1152/japplphysiol.01143.2001 11896043

[B20] Meister A Anderson ME Glutathione Annu Rev Biochem 1983 52 711 760 10.1146/annurev.bi.52.070183.003431 6137189

[B21] Dalle-Donne I Rossi R Giustarini D Colombo R Milzani A S-glutathionylation in protein redox regulation Free Radic Biol Med 2007 43 6 883 898 10.1016/j.freeradbiomed.2007.06.014 17697933

[B22] Dennis G Jr Sherman BT Hosack DA Yang J Gao W Lane HC Lempicki RA DAVID: Database for Annotation, Visualization, and Integrated Discovery Genome Biol 2003 4 5 P3 PMC193660 12734009

[B23] Huang DW Sherman BT Lempicki RA Systematic and integrative analysis of large gene lists using DAVID bioinformatics resources Nat 2009 4 1 44 57 10.1038/nprot.2008.211 19131956

[B24] Wang M You J Bemis KG Tegeler TJ Brown DPG Label-free mass spectrometry-based protein quantification technologies in proteomic analysis Brief 2008 7 5 329 339 10.1093/bfgp/eln031 18579615

[B25] Kersey PJ Duarte J Williams A Karavidopoulou Y Birney E Apweiler R The International Protein Index: an integrated database for proteomics experiments Proteomics 2004 4 7 1985 1988 10.1002/pmic.200300721 15221759

[B26] Higgs RE Knierman MD Gelfanova V Butler JP Hale JE Comprehensive label-free method for the relative quantification of proteins from biological samples J Proteome Res 2005 4 4 1442 1450 10.1021/pr050109b 16083298

[B27] Ashburner M Ball CA Blake JA Botstein D Butler H Cherry JM Davis AP Dolinski K Dwight SS Eppig JT Gene ontology: tool for the unification of biology. The Gene Ontology Consortium Nat Genet 2000 25 1 25 29 10.1038/75556 PMC3037419 10802651

[B28] Kalinina EV Chernov NN Saprin AN Involvement of thio-, peroxi-, and glutaredoxins in cellular redox-dependent processes Biochemistry (Mosc) 2008 73 13 1493 1510 10.1134/s0006297908130099 19216714

[B29] Chakravarthi S Jessop CE Bulleid NJ The role of glutathione in disulphide bond formation and endoplasmic-reticulum-generated oxidative stress EMBO Rep 2006 7 3 271 275 10.1038/sj.embor.7400645 PMC1456887 16607396

[B30] Pollak N Dolle C Ziegler M The power to reduce: pyridine nucleotides--small molecules with a multitude of functions Biochem J 2007 402 2 205 218 10.1042/BJ20061638 PMC1798440 17295611

[B31] Revollo JR Grimm AA Imai S-i The NAD biosynthesis pathway mediated by nicotinamide phosphoribosyltransferase regulates Sir2 activity in mammalian cells J Biol Chem 2004 279 49 50754 50763 10.1074/jbc.M408388200 15381699

[B32] Mosharov E Cranford MR Banerjee R The quantitatively important relationship between homocysteine metabolism and glutathione synthesis by the transsulfuration pathway and its regulation by redox changes Biochemistry 2000 39 42 13005 13011 10.1021/bi001088w 11041866

[B33] Weber G Prajda N Jackson RC Key enzymes of IMP metabolism: transformation and proliferation-linked alterations in gene expression Adv Enzyme Regul 1976 14 3 24 10.1016/0065-2571(76)90005-4 184699

[B34] Manzer R Qamar L Estey T Pappa A Petersen DR Vasiliou V Molecular cloning and baculovirus expression of the rabbit corneal aldehyde dehydrogenase (ALDH1A1) cDNA DNA Cell Biol 2003 22 5 329 338 10.1089/104454903322216671 12941160

[B35] Avval FZ Holmgren A Molecular mechanisms of thioredoxin and glutaredoxin as hydrogen donors for Mammalian s phase ribonucleotide reductase J Biol Chem 2009 284 13 8233 8240 10.1074/jbc.M809338200 PMC2659180 19176520

[B36] Herrick J Sclavi B Ribonucleotide reductase and the regulation of DNA replication: an old story and an ancient heritage Mol Microbiol 2007 63 1 22 34 10.1111/j.1365-2958.2006.05493.x 17229208

[B37] Li WW Hsiung Y Wong V Galvin K Zhou Y Shi Y Lee AS Suppression of grp78 core promoter element-mediated stress induction by the dbpA and dbpB (YB-1) cold shock domain proteins Mol Cell Biol 1997 17 1 61 68 10.1128/mcb.17.1.61 PMC231730 8972186

[B38] Shannon MF Coles LS Attema J Diamond P The role of architectural transcription factors in cytokine gene transcription J Leukoc Biol 2001 69 1 21 32 11200063

[B39] Hoogeboom D Burgering BMT Should I stay or should I go:beta-catenin decides under stress Biochim Biophys Acta 2009 1796 2 63 74 10.1016/j.bbcan.2009.02.002 19268509

[B40] Tourriere H Chebli K Zekri L Courselaud B Blanchard JM Bertrand E Tazi J The RasGAP-associated endoribonuclease G3BP assembles stress granules J Cell Biol 2003 160 6 823 831 10.1083/jcb.200212128 PMC2173781 12642610

[B41] Makeyev AV Liebhaber SA The poly(C)-binding proteins: a multiplicity of functions and a search for mechanisms Rna 2002 8 3 265 278 10.1017/s1355838202024627 PMC1370249 12003487

[B42] Dresios J Aschrafi A Owens GC Vanderklish PW Edelman GM Mauro VP Cold stress-induced protein Rbm3 binds 60S ribosomal subunits, alters microRNA levels, and enhances global protein synthesis Proc Natl Acad Sci USA 2005 102 6 1865 1870 10.1073/pnas.0409764102 PMC548588 15684048

[B43] May RC The Arp2/3 complex: a central regulator of the actin cytoskeleton Cell Mol Life Sci 2001 58 11 1607 1626 10.1007/PL00000800 PMC11337294 11706988

[B44] Stossel TP Condeelis J Cooley L Hartwig JH Noegel A Schleicher M Shapiro SS Filamins as integrators of cell mechanics and signalling Nat Rev Mol Cell Biol 2001 2 2 138 145 10.1038/35052082 11252955

[B45] Bamburg JR McGough A Ono S Putting a new twist on actin: ADF/cofilins modulate actin dynamics Trends Cell Biol 1999 9 9 364 370 10.1016/s0962-8924(99)01619-0 10461190

[B46] Hubberstey AV Mottillo EP Cyclase-associated proteins: CAPacity for linking signal transduction and actin polymerization Faseb J 2002 16 6 487 499 10.1096/fj.01-0659rev 11919151

[B47] Yu FX Lin SC Morrison-Bogorad M Atkinson MA Yin HL Thymosin beta 10 and thymosin beta 4 are both actin monomer sequestering proteins J Biol Chem 1993 268 1 502 509 8416954

[B48] Kremer BE Adang LA Macara IG Septins regulate actin organization and cell-cycle arrest through nuclear accumulation of NCK mediated by SOCS7 Cell 2007 130 5 837 850 10.1016/j.cell.2007.06.053 PMC2085444 17803907

[B49] Dransart E Olofsson B Cherfils J RhoGDIs revisited: novel roles in Rho regulation.[erratum appears in Traffic. 2006 Jan;7(1):108] Traffic 2005 6 11 957 966 10.1111/j.1600-0854.2005.00335.x 16190977

[B50] Zieger MAJ Gupta MP Endothelial cell preservation at 10 degrees C minimizes catalytic iron, oxidative stress, and cold-induced injury Cell Transplant 2006 15 6 499 510 10.3727/000000006783981756 17121161

[B51] Zieger MAJ Gupta MP Siddiqui RA Endothelial cell fatty acid unsaturation mediates cold-induced oxidative stress J Cell Biochem 2006 99 3 784 796 10.1002/jcb.20961 16676360

[B52] Gupta MP Zieger MA Proteomic Analysis of Hypothermically Preconditioned Endothelial Cells Reveals Comprehensive Protection from Hypothermic Injury The FASEB Journal 2006 20 5 A828

[B53] Brinkkoetter P-T Song H Losel R Schnetzke U Gottmann U Feng Y Hanusch C Beck GC Schnuelle P Wehling M van der Woude FJ Yard BA Hypothermic injury: the mitochondrial calcium, ATP and ROS love-hate triangle out of balance Cell Physiol Biochem 2008 22 1-4 195 204 10.1159/000149797 18769046

[B54] Persa C Pierce A Ma Z Kabil O Lou MF The presence of a transsulfuration pathway in the lens: a new oxidative stress defense system Experimental Eye Research 2004 79 6 875 886 10.1016/j.exer.2004.06.029 15642325

[B55] Townsend DM Manevich Y He L Hutchens S Pazoles CJ Tew KD Novel role for glutathione S-transferase pi. Regulator of protein S-Glutathionylation following oxidative and nitrosative stress J Biol Chem 2009 284 1 436 445 10.1074/jbc.M805586200 PMC2610519 18990698

[B56] Schuppe-Koistinen I Gerdes R Moldeus P Cotgreave IA Studies on the reversibility of protein S-thiolation in human endothelial cells Arch Biochem Biophys 1994 315 2 226 234 10.1006/abbi.1994.1494 7986062

[B57] Ralser M Wamelink M Kowald A Gerisch B Heeren G Struys E Klipp E Jakobs C Breitenbach M Lehrach H Krobitsch S Dynamic rerouting of the carbohydrate flux is key to counteracting oxidative stress Journal of Biology 2007 6 4 10 10.1186/jbiol61 PMC2373902 18154684

[B58] Jayaram HN Dion RL Glazer RI Johns DG Robins RK Srivastava PC Cooney DA Initial studies on the mechanism of action of a new oncolytic thiazole nucleoside, 2-beta-D-ribofuranosylthiazole-4-carboxamide (NSC 286193) Biochem Pharmacol 1982 31 14 2371 2380 10.1016/0006-2952(82)90532-9 6127085

[B59] Saladino CF Johnson HA Rate of DNA synthesis as a function of temperature in cultured hamster fibroblasts (V-79) and HeLa-S3 cells Exp Cell Res 1974 85 2 248 254 10.1016/0014-4827(74)90124-4 4857155

[B60] Stryer L Biochemistry 1975 San Francisco: W. H. Freeman and Company 290 291

[B61] Hagopian K Ramsey JJ Weindruch R Enzymes of glycerol and glyceraldehyde metabolism in mouse liver: effects of caloric restriction and age on activities Biosci Rep 2008 28 2 107 115 10.1042/BSR20080015 PMC2538689 18429748

[B62] Hibuse T Maeda N Nakatsuji H Tochino Y Fujita K Kihara S Funahashi T Shimomura I The heart requires glycerol as an energy substrate through aquaporin 7, a glycerol facilitator Cardiovascular Research 2009 83 1 34 41 10.1093/cvr/cvp095 19297367

[B63] Gambert S Helies-Toussaint C Grynberg A Extracellular glycerol regulates the cardiac energy balance in a working rat heart model Am J Physiol Heart Circ Physiol 2007 292 3 H1600 1606 10.1152/ajpheart.00563.2006 17040970

[B64] Gambert S Héliès-Toussaint C Grynberg A Regulation of intermediary metabolism in rat cardiac myocyte by extracellular glycerol Biochimica et Biophysica Acta (BBA) - Molecular and Cell Biology of Lipids 2005 1736 2 152 162 10.1016/j.bbalip.2005.08.004 16153888

[B65] Nguyen NHT Bråthe A Hassel B Neuronal uptake and metabolism of glycerol and the neuronal expression of mitochondrial glycerol-3-phosphate dehydrogenase Journal of Neurochemistry 2003 85 4 831 842 10.1046/j.1471-4159.2003.01762.x 12716415

[B66] Jeong D-w Kim T-S Cho IT Kim IY Modification of glycolysis affects cell sensitivity to apoptosis induced by oxidative stress and mediated by mitochondria Biochemical and Biophysical Research Communications 2004 313 4 984 991 10.1016/j.bbrc.2003.12.033 14706639

[B67] Serfling E Berberich-Siebelt F Avots A Chuvpilo S Klein-Hessling S Jha MK Kondo E Pagel P Schulze-Luehrmann J Palmetshofer A NFAT and NF-kappaB factors-the distant relatives Int J Biochem Cell Biol 2004 36 7 1166 1170 10.1016/j.biocel.2003.07.002 15109564

[B68] Yan C Boyd DD Histone H3 acetylation and H3 K4 methylation define distinct chromatin regions permissive for transgene expression Mol Cell Biol 2006 26 17 6357 6371 10.1128/MCB.00311-06 PMC1592829 16914722

[B69] Eirin-Lopez JM Gonzalez-Romero R Dryhurst D Ishibashi T Ausio J The evolutionary differentiation of two histone H2A.Z variants in chordates (H2A.Z-1 and H2A.Z-2) is mediated by a stepwise mutation process that affects three amino acid residues BMC Evol Biol 2009 9 31 10.1186/1471-2148-9-31 PMC2644675 19193230

[B70] Kumar SV Wigge PA H2A.Z-containing nucleosomes mediate the thermosensory response in Arabidopsis Cell 140 1 136 147 10.1016/j.cell.2009.11.006 20079334

[B71] Cedar H Bergman Y Linking DNA methylation and histone modification: patterns and paradigms Nat Rev Genet 2009 10 5 295 304 10.1038/nrg2540 19308066

[B72] James SJ Melnyk S Pogribna M Pogribny IP Caudill MA Elevation in S-adenosylhomocysteine and DNA hypomethylation: potential epigenetic mechanism for homocysteine-related pathology J Nutr 2002 132 8 Suppl 2361S 2366S 10.1093/jn/132.8.2361S 12163693

[B73] Kalushkova A Fryknas M Lemaire M Fristedt C Agarwal P Eriksson M Deleu S Atadja P Osterborg A Nilsson K Vanderkerken K Oberg F Jernberg-Wiklund H Polycomb target genes are silenced in multiple myeloma PLoS ONE 5 7 e11483 10.1371/journal.pone.0011483 PMC2901331 20634887

[B74] Anderson RM Bitterman KJ Wood JG Medvedik O Cohen H Lin SS Manchester JK Gordon JI Sinclair DA Manipulation of a nuclear NAD+ salvage pathway delays aging without altering steady-state NAD+ levels J Biol Chem 2002 277 21 18881 18890 10.1074/jbc.M111773200 11884393

[B75] van der Veer E Ho C O'Neil C Barbosa N Scott R Cregan SP Pickering JG Extension of human cell lifespan by nicotinamide phosphoribosyltransferase J Biol Chem 2007 282 15 10841 10845 10.1074/jbc.C700018200 17307730

[B76] Yang T Sauve AA NAD metabolism and sirtuins: metabolic regulation of protein deacetylation in stress and toxicity Aaps J 2006 8 4 E632 643 10.1208/aapsj080472 PMC2751359 17233528

[B77] Sedoris KC Thomas SD Miller DM c-myc promoter binding protein regulates the cellular response to an altered glucose concentration Biochemistry 2007 46 29 8659 8668 10.1021/bi7003558 17595061

[B78] Hurlin PJ N-Myc functions in transcription and development Birth Defects Res Part C Embryo Today 2005 75 4 340 352 10.1002/bdrc.20059 16425253

[B79] Rutkowski DT Kaufman RJ A trip to the ER: coping with stress Trends Cell Biol 2004 14 1 20 28 10.1016/j.tcb.2003.11.001 14729177

[B80] Mamady H Storey KB Coping with the stress: expression of ATF4, ATF6, and downstream targets in organs of hibernating ground squirrels Arch Biochem Biophys 2008 477 1 77 85 10.1016/j.abb.2008.05.006 18541136

[B81] Cullinan SB Diehl JA PERK-dependent activation of Nrf2 contributes to redox homeostasis and cell survival following endoplasmic reticulum stress J Biol Chem 2004 279 19 20108 20117 10.1074/jbc.M314219200 14978030

[B82] Harding HP Zhang Y Zeng H Novoa I Lu PD Calfon M Sadri N Yun C Popko B Paules R Stojdl DF Bell JC Hettmann T Leiden JM Ron D An integrated stress response regulates amino acid metabolism and resistance to oxidative stress Mol Cell 2003 11 3 619 633 10.1016/s1097-2765(03)00105-9 12667446

[B83] Deneke SM Baxter DF Phelps DT Fanburg BL Increase in endothelial cell glutathione and precursor amino acid uptake by diethyl maleate and hyperoxia Am J Physiol 1989 257 4 Pt 1 L265 271 10.1152/ajplung.1989.257.4.L265 2801955

[B84] Matsumoto K Wolffe AP Gene regulation by Y-box proteins: coupling control of transcription and translation Trends Cell Biol 1998 8 8 318 323 10.1016/s0962-8924(98)01300-2 9704408

[B85] Ermolenko DN Makhatadze GI Bacterial cold-shock proteins Cell Mol Life Sci 2002 59 11 1902 1913 10.1007/PL00012513 PMC11337450 12530521

[B86] Mackintosh C Dynamic interactions between 14-3-3 proteins and phosphoproteins regulate diverse cellular processes Biochem J 2004 381 Pt 2 329 342 10.1042/BJ20031332 PMC1133837 15167810

[B87] Szopa J Transgenic 14-3-3 isoforms in plants: the metabolite profiling of repressed 14-3-3 protein synthesis in transgenic potato plants Biochem Soc Trans 2002 30 4 405 410 10.1042/bst0300405 12196104

[B88] Desai A Mitchison TJ Microtubule polymerization dynamics Annu Rev Cell Dev Biol 1997 13 83 117 10.1146/annurev.cellbio.13.1.83 9442869

[B89] Sapir T Elbaum M Reiner O Reduction of microtubule catastrophe events by LIS1, platelet-activating factor acetylhydrolase subunit Embo J 1997 16 23 6977 6984 10.1093/emboj/16.23.6977 PMC1170301 9384577

[B90] Gourlay CW Ayscough KR The actin cytoskeleton: a key regulator of apoptosis and ageing? Nat Rev Mol Cell Biol 2005 6 7 583 589 10.1038/nrm1682 16072039

[B91] Ramchandran R Mehta D Vogel SM Mirza MK Kouklis P Malik AB Critical role of Cdc42 in mediating endothelial barrier protection in vivo Am J Physiol Lung Cell Mol Physiol 2008 295 2 L363 369 10.1152/ajplung.90241.2008 PMC2519844 18515405

[B92] Nobes CD Hall A Rho, rac, and cdc42 GTPases regulate the assembly of multimolecular focal complexes associated with actin stress fibers, lamellipodia, and filopodia Cell 1995 81 1 53 62 10.1016/0092-8674(95)90370-4 7536630

[B93] Vignjevic D Kojima S-i Aratyn Y Danciu O Svitkina T Borisy GG Role of fascin in filopodial protrusion J Cell Biol 2006 174 6 863 875 10.1083/jcb.200603013 PMC2064340 16966425

[B94] Faix J Rottner K The making of filopodia Curr Opin Cell Biol 2006 18 1 18 25 10.1016/j.ceb.2005.11.002 16337369

[B95] Kureishy N Sapountzi V Prag S Anilkumar N Adams JC Fascins, and their roles in cell structure and function Bioessays 2002 24 4 350 361 10.1002/bies.10070 11948621

[B96] Suzuki T Nakamoto T Ogawa S Seo S Matsumura T Tachibana K Morimoto C Hirai H MICAL, a novel CasL interacting molecule, associates with vimentin J Biol Chem 2002 277 17 14933 14941 10.1074/jbc.M111842200 11827972

[B97] Le Clainche C Carlier M-F Regulation of actin assembly associated with protrusion and adhesion in cell migration Physiol Rev 2008 88 2 489 513 10.1152/physrev.00021.2007 18391171

[B98] Wiche G Role of plectin in cytoskeleton organization and dynamics J Cell Sci 1998 111 Pt 17 2477 2486 10.1242/jcs.111.17.2477 9701547

[B99] Moser M Nieswandt B Ussar S Pozgajova M Fassler R Kindlin-3 is essential for integrin activation and platelet aggregation Nat Med 2008 14 3 325 330 10.1038/nm1722 18278053

[B100] Prasain N Stevens T The actin cytoskeleton in endothelial cell phenotypes Microvasc Res 2009 77 1 53 63 10.1016/j.mvr.2008.09.012 PMC2700738 19028505

[B101] Noren NK Pasquale EB Eph receptor-ephrin bidirectional signals that target Ras and Rho proteins Cell Signal 2004 16 6 655 666 10.1016/j.cellsig.2003.10.006 15093606

[B102] Hazel JR Effects of temperature on the structure and metabolism of cell membranes in fish Am J Physiol 1984 246 4 Pt 2 R460 470 10.1152/ajpregu.1984.246.4.R460 6372513

[B103] Zhang XH Zhao C Ma ZA The increase of cell-membranous phosphatidylcholines containing polyunsaturated fatty acid residues induces phosphorylation of p53 through activation of ATR J Cell Sci 2007 120 Pt 23 4134 4143 10.1242/jcs.015834 PMC2915541 18032786

[B104] Kim JS Southard JH Phospholipid metabolism of hypothermically stored rat hepatocytes Hepatology 1999 30 5 1232 1240 10.1002/hep.510300531 10534345

[B105] Andersson DA Nash M Bevan S Modulation of the cold-activated channel TRPM8 by lysophospholipids and polyunsaturated fatty acids J Neurosci 2007 27 12 3347 3355 10.1523/JNEUROSCI.4846-06.2007 PMC2726637 17376995

[B106] Imai A Takahashi M Nozawa Y Phospholipid metabolism in human platelets preserved at 22 degrees C: differential effects of storage on phospholipase A2- and C-mediated reactions Cryobiology 1984 21 3 255 259 10.1016/0011-2240(84)90321-3 6734238

[B107] Choe M Jackson C Yu BP Lipid peroxidation contributes to age-related membrane rigidity Free Radic Biol Med 1995 18 6 977 984 10.1016/0891-5849(94)00217-8 7628733

[B108] Chen JJ Yu BP Alterations in mitochondrial membrane fluidity by lipid peroxidation products Free Radic Biol Med 1994 17 5 411 418 10.1016/0891-5849(94)90167-8 7835747

[B109] Higgs RE Knierman MD Freeman AB Gelbert LM Patil ST Hale JE Estimating the statistical significance of peptide identifications from shotgun proteomics experiments J Proteome Res 2007 6 5 1758 1767 10.1021/pr0605320 17397207

[B110] Fitzpatrick DPG You J Bemis KG Wery J Ludwig JR Wang M Searching for potential biomarkers of cisplatin resistance in human ovarian cancer using a label-free LC/MS-based protein quantification method Proteomics Clin Appl 2007 1 246 263 10.1002/prca.200600768 21136676

[B111] Bantscheff M Schirle M Sweetman G Rick J Kuster B Quantitative mass spectrometry in proteomics: a critical review Anal Bioanal Chem 2007 389 4 1017 1031 10.1007/s00216-007-1486-6 17668192

[B112] Baik JY Lee MS An SR Yoon SK Joo EJ Kim YH Park HW Lee GM Initial transcriptome and proteome analyses of low culture temperature-induced expression in CHO cells producing erythropoietin Biotechnol Bioeng 2006 93 2 361 371 10.1002/bit.20717 16187333

[B113] Kumar N Gammell P Meleady P Henry M Clynes M Differential protein expression following low temperature culture of suspension CHO-K1 cells BMC Biotechnol 2008 8 42 10.1186/1472-6750-8-42 PMC2386802 18430238

[B114] Hale JE Butler JP Gelfanova V You J-S Knierman MD A simplified procedure for the reduction and alkylation of cysteine residues in proteins prior to proteolytic digestion and mass spectral analysis Anal Biochem 2004 333 1 174 181 10.1016/j.ab.2004.04.013 15351294

[B115] Nagy L Nagata M Szabo S Protein and non-protein sulfhydryls and disulfides in gastric mucosa and liver after gastrotoxic chemicals and sucralfate: possible new targets of pharmacologic agents World J Gastroenterol 2007 13 14 2053 2060 10.3748/wjg.v13.i14.2053 PMC4319124 17465447

[B116] Ishii Y Partridge CA Del Vecchio PJ Malik AB Tumor necrosis factor-alpha-mediated decrease in glutathione increases the sensitivity of pulmonary vascular endothelial cells to H2O2 J Clin Invest 1992 89 3 794 802 10.1172/JCI115658 PMC442924 1541673

